# Tangential Intrahypothalamic Migration of the Mouse Ventral Premamillary Nucleus and Fgf8 Signaling

**DOI:** 10.3389/fcell.2021.676121

**Published:** 2021-05-19

**Authors:** Lara López-González, Antonia Alonso, Elena García-Calero, Eduardo de Puelles, Luis Puelles

**Affiliations:** ^1^Department of Human Anatomy and Psychobiology, School of Medicine, University of Murcia, Murcia, Spain; ^2^Biomedical Research Institute of Murcia (IMIB-Arrixaca), Murcia, Spain; ^3^Instituto de Neurociencias de Alicante, CSIC, Universidad Miguel Hernández, Alicante, Spain

**Keywords:** neuronal tangential migration, ventral premamillary nucleus (VPM), Fgf8, hypothalamus, organotypic cultures, retromamillary area (RM), dorsal premamillary nucleus (DPM), perimamillary band

## Abstract

The tuberal hypothalamic ventral premamillary nucleus (VPM) described in mammals links olfactory and metabolic cues with mating behavior and is involved in the onset of puberty. We offer here descriptive and experimental evidence on a migratory phase in the development of this structure in mice at E12.5–E13.5. Its cells originate at the retromamillary area (RM) and then migrate tangentially rostralward, eschewing the mamillary body, and crossing the molecularly distinct perimamillary band, until they reach a definitive relatively superficial ventral tuberal location. Corroborating recent transcriptomic studies reporting a variety of adult glutamatergic cell types in the VPM, and different projections in the adult, we found that part of this population heterogeneity emerges already early in development, during tangential migration, in the form of differential gene expression properties of at least 2–3 mixed populations possibly derived from subtly different parts of the RM. These partly distribute differentially in the core and shell parts of the final VPM. Since there is a neighboring acroterminal source of Fgf8, and Fgfr2 is expressed at the early RM, we evaluated a possible influence of Fgf8 signal on VPM development using hypomorphic Fgf8^neo/null^ embryos. These results suggested a trophic role of Fgf8 on RM and all cells migrating tangentially out of this area (VPM and the subthalamic nucleus), leading in hypomorphs to reduced cellularity after E15.5 without alteration of the migrations proper.

## Introduction

The hypothalamic tuberal ventral premamillary nucleus (VPM) was first identified by Gurdjian (1927). Canteras et al. (1992) and Merlino et al. (2019) studied its projections, which target the periaqueductal gray, the lateral tuberal area, the paraventricular hypothalamic nucleus, the preoptic area, various medial paraseptal BST nuclei, the ventral lateral septal nucleus and the amygdalo-hippocampal amygdalar nucleus. The VPM receives inputs from forebrain structures related to the vomeronasal system, conveying conspecific and heterospecific olfactory signals (Cavalcante et al., 2014). This nucleus apparently links signals of somatic energy balance and olfaction with mating behavior (Donato and Elias, 2011).

**FIGURE 1:**
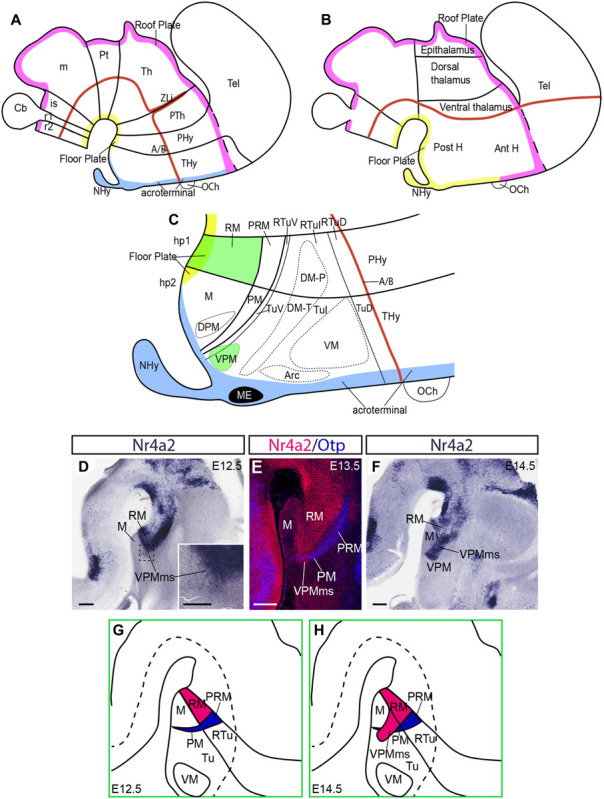
Hypothalamus in the prosomeric model and a RM population invading hp2. **(A,B)** Sketches of the prosomeric and columnar models, emphasizing differences in the concept of the axis (red line, representing also alar-basal boundary) and longitudinal floor and roof zones (yellow and pink lines, respectively). Blue denotes the acroterminal domain, not distinguished in the columnar model. **(C)** More detailed schema of the basal hypothalamus in the prosomeric model, centered upon the postulated progenitor areas. The RM area and the migrated VPM nucleus are drawn in green. **(D–H)** Representative images and schemas of our preliminary molecular evidence and interpretive analysis of the VPM origin and migration out of RM; **(D–F)** three *Nr4a2*-labeled sagittal sections of E12.5, E13.5, and E14.5 embryos showing progressive emergence of a positive cell group out of the RM area (VPMms), leading to the VPM. **(D,F)** *In situ* reaction. **(E)** This dark-field microphotograph shows two-color immunoreaction for Nr4a2 (red) and Otp, the latter identifying the PM/PRM band (blue; compare with **C**). **(G,H)** Schemata of the emergence of the Nr4a2-positive rostrally migrating RM population relative to neighboring areas. Scale bars represent 200 μm in **(D–F)**, and 100 μm in **(D)** inset.

The VPM appears as a relatively superficial ovoidal cell aggregate intercalated between the mamillary body and the hypophysial infundibulum, halfway between the ventromedial hypothalamic nucleus and the mamillary nucleus. It is separated from the latter by the histaminergic tuberomamillary area and the perimamillary band (the latter contains the conventional dorsal premamillary nucleus; DPM; Figure 1C). Its rostral infundibular relationship abuts the acroterminal hypothalamic domain (rostral end of the hypothalamus in the prosomeric model; Puelles et al., 2012; Puelles and Rubenstein, 2015; Diaz and Puelles, 2020). The VPM contains excitatory glutamatergic neurons, which display a diversity of molecular profiles, possibly related to its different projections (Ziegler et al., 2002; Mickelsen et al., 2020). A field rich in inhibitory neurons surrounds the VPM, which corresponds to the tuberal terminal part of the dorsomedial hypothalamic nucleus (DM-T; Figure 1C; Puelles et al., 2012). In prosomeric terms, its adult position is within the tuberal intermediate region in the basal plate of the terminal hypothalamus, dorsally to the topologically subjacent perimamillary and mamillary regions (PM; M; Figures 1C,E,G,H; Puelles et al., 2012).

A needed note of caution is that the ‘ventral’ and ‘premamillary’ descriptors present in the conventional VPM name obey to their origin and usage within the columnar schema of the diencephalon; they refer to a different forebrain axis (Figure 1B; Herrick, 1910, 1948; Kuhlenbeck, 1973; the same happens with the DPM). These descriptors are misnomers in the prosomeric conception (Figure 1A). The VPM is dorsal to the perimamillary DPM and the mamillary complex in our model. Neither is ‘premamillary,’ because in our model there is nothing more rostral than the acroterminal part of the mamillary area (Figures 1A,C). We will use henceforth the axial reference and descriptive terminology of the prosomeric model (Figures 1A,C), without changing the conventional names of the nuclei.

It is remarkable that VPM neurons do not reproduce the molecular profile of the intermediate tuberal hypothalamic domain where they reside (gene markers such as *Dlk1*, *Nkx2.1*, *Dlx5-6*, *Rgs4*, *Zfhx3*, or *Efna5* are widely expressed), nor those of the perimamillary or mamillary domains underneath. In contrast, a number of embryonic markers label selectively VPM (including *Foxa1*, *Nr4a2*, *Irx1*, *Irx5*, *Enc1*, *Lmx1b*, *Nos1*, *Pknox2*, absent otherwise at the ventral tuberal and intermediate tuberal areas), and, surprisingly, these are also present at the retromamillary hypothalamic basal microzone, which is the ventralmost basal component of the peduncular hypothalamus, and the neuromeric neighbor immediately caudal to the mamillary body (RM; green background in Figure 1C; Shimogori et al., 2010; Puelles et al., 2012); see also Supplementary Table 2. A differential molecular profile of various postnatal VPM cell types was described recently (Mickelsen et al., 2020). These authors studied markers that start to be expressed at late embryonic or postnatal stages (according to the Allen Developing Mouse Brain Atlas; www.developingmouse.brain-map.org). In this report we study earlier phenomena characterized by developmental gene markers.

The present report was inspired by insights offered by Puelles et al. (2012; see their p. 293 and Figures 8.31, 8.32), based on embryonic *Irx5*, *Nos1*, *Pknox2*, and *Tac1* expression analysis. These authors suggested that the VPM neuronal population possibly originates in the RM (thus explaining the similarity in molecular profile), and subsequently migrates tangentially into ventral tuberal and intermediate tuberal areas, crossing obliquely the PM band, to constitute the definitive VPM nucleus (see sketch of the postulated migration path in Figure 1H).

The aim of the present report was to check this hypothesis. We examined additional sequential gene expression data (notably *Foxa1* and *Nr4a2* as selective VPM markers, and *Otp*; *Sim1*; *Ebf3*; *Nkx2.1*; *Fezf2* as reference markers). Further, we performed CMFDA fluorescent *in vitro* labeling experiments at the presumed source area of the migration (RM), as well as along its path and control sites, in organotypic forebrain explants. These data jointly corroborated the cited migratory hypothesis and illuminated other unknown aspects of the phenomenon. In addition, given that the migrated VPM abuts the hypothalamic basal acroterminal domain (the latter appears in blue in Figure 1C), and there is evidence of *Fgf8* expression at this rostral site and *Fgfr2* expression at the RM (Allen Developing Mouse Brain Atlas), we analyzed the possible role of basal acroterminal FGF8 morphogen spreading out caudalwards, which might act as an attractor upon the VPM migration. To this end we studied 2 specimens of severe hypomorph Fgf8 mice, known to express significantly reduced levels of the secreted morphogen protein FGF8 (Fgf8^neo/null^; Meyers et al., 1998). These embryos showed a hypotrophic RM and VPM phenotype.

## Materials and Methods

### Animals

E15.5 and E18.5 Fgf8^neo/null^ embryos and wildtype controls from the same litter were kindly provided by E. de Puelles (Alicante Institute of Neuroscience, Experimental Embryology group).

Fgf8^neo/+^ and Fgf8^null/+^ mice were crossed to obtain severe hypomorph Fgf8^null/neo^ mutants (C57BL/6 genetic background). PCR genotyping was performed as described by Chi et al. (2003).

The morning in which a vaginal plug was detected was considered as E0.5 in all mice. Brains destined to *in situ* hybridization (ISH), immunofluorescence or immunohistochemistry were fixed overnight with 4% paraformaldehyde in phosphate saline buffer (PBS) at 4°C. After washing, they were embedded in 2% low viscosity Agarose in PBS. Vibratome sections were obtained (100 μm-thick for ISH –with or without counterstain- and 50 μm-thick for immunoreactions).

### Immunohistochemistry

For immunofluorescent staining, the vibratome sections were blocked in PBS containing 0.3% Triton X-100 (PBT) and 3% BSA (bovine serum albumin), after several washes in PBT. The sections were incubated in the primary antibodies for 48 h at 4°C, using at the following concentrations: goat anti Nr4a2 (1:200; AF2156, RyD Systems), rabbit anti Foxa1 (1:200; ab23738, Abcam), rabbit anti Otp (1:50; kindly provided by A. Simeone). Following incubation and several PBT washes, the sections were incubated 2 h with the respective fluorochrome-labeled secondary antibodies (1:200; Donkey anti-goat Alexa 594, Donkey anti-rabbit Alexa 488, or Donkey anti rabbit Alexa 647, as required; Thermo Fisher Scientific).

For conventional immunohistochemistry (IHC), sections were washed in PBS and then treated with 0.1% hydrogen peroxide in PBS for 1 h in the dark to inactivate endogenous peroxidase activity. After standard PBT washes and blocking steps, floating sections were incubated with the primary antibody rabbit anti Foxa1 (1:200; ab23738, Abcam) for 48 h at 4°C. After PBT washes we applied a biotinylated goat anti rabbit secondary antibody (1:200, 2 h; Vector Laboratories, Burlingame, CA, United States), and thereafter a streptavidin/horseradish peroxidase (HRP) complex (1:200, 2 h of incubation; Vectastain-ABC kit; Vector Laboratories, Burlingame, CA, United States). Histochemical detection of the peroxidase activity was carried out using 0.03% diaminobenzidine (DAB) and 0.005% H2O2.

### *In situ* Hybridization

The hybridization protocol used was according to Shimamura et al. (1994). The riboprobes used were *Ebf3*, *Fezf2*, *Foxa1*, *Foxb1*, *Nr4a2*, and *Sim1* (Supplementary Table 1).

### Organotypic Culture

Brains of embryos extracted at E11.5–E14.5 were collected in ice-cold artificial cerebrospinal fluid medium (pH 7.4), containing: 4 mM KCl, 1.5 CaCl_2_, 0.75 mM MgCl_2_, 129 mM NaCl, and 10 mM D-glucose. We dissected the tissue partially, discarding meninges and the telencephalic vesicles, and opened the neural tube along the midline with watchmaker forceps. We placed separately the two brain halves upon membrane culture inserts (Millicell Millipore, 0.4 μm, PICM0RG50) within small Petri dishes, with the ventricular surface up, contacting the air, and the pial surface touching across the membrane a substrate of MEM-supplemented medium (with added 1% PenStrep, 0.065% glucose, 0.5% glutamine, and complement inactivated 1% fetal bovine serum). The explants were acclimatized for 1 h under culture conditions (5% CO_2_, 37°C), and then were marked with tungsten microcarriers (BioRad, #165229) coated with CMFDA (Termofisher, C2925), which were transferred with a sharpened tungsten needle to chosen points of the RM, following the Alifragis et al. (2002) protocol. After the labeling, the medium was changed to supplemented Neurobasal medium (with added 1% PenStrep, 0.065% glucose, 0.5% glutamine, and 1% B27 supplement). The explants were incubated for 2 days under standard conditions (5% CO_2_, 37°C). The cultures were then fixed in cold 4% PF in PBS for 10 min, and were processed for immunofluorescence (IF), following the protocol previously described.

To assess the positioning of the grain of CMFDA consistently in the diverse specimens, we created a first dorsoventral division of the full RM into its dorsal and ventral halves. Most experiments concentrated on the dorsal half of RM, due to the results of preliminary experiments indicating that little migration was obtained from the ventral half of RM. The dorsal half of RM was divided further dorsoventrally into three nearly equal-sized longitudinal parts, and these were subdivided anteroposteriorly into three transversal parts. The resulting four dorsoventral subdivisions were named numerically from dorsal to ventral (1–4), while the transversal subdivisions were identified as caudal, middle, and rostral subregions (C, M, R).

### Image Analysis

We scanned the ISH and ISH/IHC images at high resolution with Aperio ImageScope software (Leica Biosystems), and adjusted brightness and contrast for publication with Photoshop software (Adobe). We obtained IF images from fixed sections and cultured explants using a confocal SP8 Leica microscope. Individual optic sections were 3 μm apart, and image stacks of various Z sizes were generated according to the structures of interest. Figures were constructed using Adobe Photoshop and Adobe Ilustrator software.

## Results

Figure 1C schematizes the position of VPM within the overall areal structure of the hypothalamic basal plate postulated in the updated prosomeric model (Puelles et al., 2012; Puelles and Rubenstein, 2015; see also Puelles, 1995, 2001, 2013, 2018 for general comparison with other models). This model defines two hypothalamo-telencephalic prosomeres, hp1 and hp2 (Figure 1C; note their caudorostral order); hp2 ends at the forebrain’s rostromedian midline in a singular acroterminal domain (blue in Figures 1A,C; Puelles et al., 2012; Puelles and Rubenstein, 2015). The respective hypothalamic regions comprise the peduncular and terminal hypothalamus subregions, respectively (PHy; THy; Figures 1A,C; Puelles et al., 2012). Both peduncular hypothalamus and terminal hypothalamus territories (including the acroterminal domain) are subdivided into alar and basal portions that are in caudal continuity with correlative diencephalic longitudinal subdivisions (Puelles et al., 2012; Morales-Delgado et al., 2014; Ferran et al., 2015; Puelles and Rubenstein, 2015).

The RM area forms the ventralmost basal subdomain within the peduncular hypothalamus, a part of the hp1 prosomere; i.e., RM lies adjacent to the floor plate (PHy; RM in green; Figure 1C). RM contacts rostrally with the mamillary area, which belongs to the ventralmost basal domain of the terminal hypothalamus, a part of the hp2 prosomere (M; THy; Figure 1C; Puelles et al., 2012). In this model, RM and M jointly (RM/M) represent the ventralmost longitudinal subdomain of the basal hypothalamus next to the hypothalamic floor plate (note the traditional columnar interpretation is quite different; RM/M are described instead as ‘posterior hypothalamic’ structures; compare Figures 1A,B). During early development, the RM and M subdomains are identifiable once neurogenesis begins, both by the bilateral large external bulge of the massive mamillary body, distinguishable as of E13.5–E14.5, and by their differential gene expression patterns: *Foxb1*, *Nkx2.1*, *Unc5b*, *Nhlh2*, and *Sim1* are selectively expressed in the mamillary body, in contrast with the VPM markers mentioned above, also expressed in the RM area (*Foxa1*, *Nr4a2*, *Irx1*, *Irx5*, *Enc1*, *Lmx1b*, *Nos1*, *Pknox2*; Supplementary Table 2).

The basal region of the hypothalamus displays dorsally to RM/M two other molecularly distinct longitudinal territories: the rostrally tapering periretromamillary/perimamillary band (PRM continuous with PM; Figures 1C,E,G,H). This band is defined by the selective expression of *Otp*, *Otx*, *Zic1*, and *Fezf2* genes. It shares *Sim1* signal with M, and *Ebf3* with RM. Dorsal to PRM/PM there are the corresponding larger retrotuberal and tuberal basal regions (RTu continuous with Tu; Figures 1C,G,H). This large territory is further subdivided cytoarchitectonically and molecularly into three dorsoventrally disposed longitudinal subregions. The terminal ones are identified as dorsal, intermediate, and ventral Tu subdomains (TuD, TuI, TuV; Figure 1C; Puelles et al., 2012), and they are continuous caudalward with corresponding peduncular RTu subdomains (RTuD, RTuI, RTuV; Figure 1C). The VPM is found in the adult mouse between the ventral tuberal area and a ventrorostral part of the intermediate tuberal area (under the ventromedial hypothalamic nucleus). The thin ventral tuberal subdomain (TuV; or tuberomamillary area), partly invaded by the VPM, is where histaminergic neurons are produced (TuV; Figure 1C; Puelles et al., 2012).

Our analysis of the VPM migration is done according to the updated prosomeric model, wherein the forebrain axis bends into the hypothalamus following the cephalic flexure, ending at the acroterminal midline (Figures 1A,C; Puelles et al., 2012, 2013; Puelles and Rubenstein, 2015). This axis courses (jointly with the related alar-basal boundary) parallel to the RM/M floor plate, held to be induced by the rostral tip of the notochord (García-Calero et al., 2008; Puelles et al., 2012).

### The VPM Migration Stream Originates at the RM and Ends Forming the VPM Nucleus

We searched for VPM gene markers at E13.5, E15.5, E18.5 and P14 with the aid of the AGEA tool of the Allen Developing Mouse Brain Atlas https://developingmouse.brain-map.org/. Most of the 22 VPM genes selected (Supplementary Table 2) are shared by VPM and RM (14 at E13.5, and 16 at E15.5). Other genes not expressed in RM (e.g., *Ar*, *Foxp1*, *LepR*, *Tac1*) appear belatedly in VPM (see also additional late markers in Mickelsen et al., 2020).

We also analyzed gene markers shared by VPM with the subthalamic nucleus complex (STh), and the dorsal premamillary nucleus (DPM). Some early VPM-RM genes mark these three neighboring structures (e.g., *Bcl11a* and *Grik2*). Other genes such as *Foxa1*, *Lmx1a*, *Lmx1b*, or *Pbx3* mark both VPM and STh, but not the DPM. Contrarily, *Nr4a2* marks VPM and DPM, but not STh.

**FIGURE 2:**
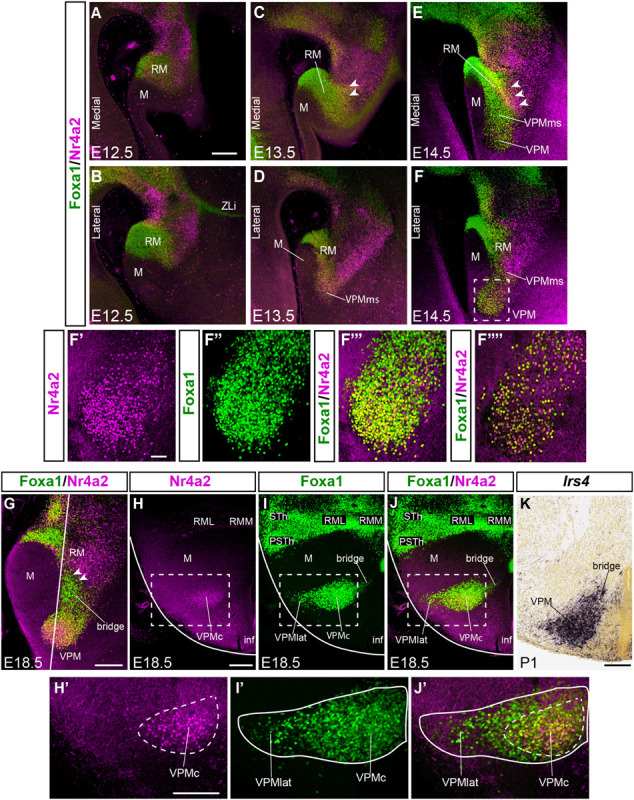
Comparison of Foxa1 and Nr4a2 signal during VPM development. **(A–F)** Double immunofluorescence of Foxa1 (green) and Nr4a2 (magenta) in medial **(A,C,E)** and lateral **(B,D,F)** sagittal sections of E12.5, E13.5, and E14.5 mice embryos, showing RM, M, VPMms, and the prospective VPM. Distinct ventral Foxa1 and dorsal Nr4a2 RM domains are observed, with an intermediate yellow (double-labeled) subpopulation, best visible in **(C,E)** (white arrowheads). **(F’–F”’)** High magnification images of the square area dashed around VPM in **(F)** showing the separate magenta and green channels **(F’,F”)** and their conjunction **(F”’)** thinner, with apparently double-labeled cells. These are confocal reconstructions of a 50 mm-thick stack of 3 μm optical slices. The image in **(F””)** shows a similar, but only 3 mm-thick stack, indicating that double-labeled cells really exist. **(G)** Sagittal section at E18.5 illustrating the differential distribution of Foxa1 and Nr4a2 cells within RM, VPM, and the RM-VPM connecting bridge, ventrally composed by Foxa1 cells (white arrowheads). The white line indicates the plane of section used for panels **(H–J)**. **(H–J)** Horizontal sections showing the differential medio-lateral distribution Foxa1 and Nr4a2 VPM cells at E18.5. **(H,H’)** Nr4a2 marks RM and the core of VPM -VPMc. **(I,I’,J,J’)** Foxa1 labels the RM, lateral (superficial shell) and core parts of VPM -VPMlat, VPMc-, as well as the separate STh and PSTh migrated populations. **(K)** This horizontal section similar to those in **(H–J)** but labeled with *Irs4* ISH at P1 shows the VPM and its persistent bridge. Scale bar in **(A)** represents 200 μm, valid also for **(B–F)**. Scale bars in **(G,H)** represent 200 μm (valid also for **I–K**). Scale bar in **(F’)** represents 75 μm (valid also for **F”,F”’**).

**FIGURE 3:**
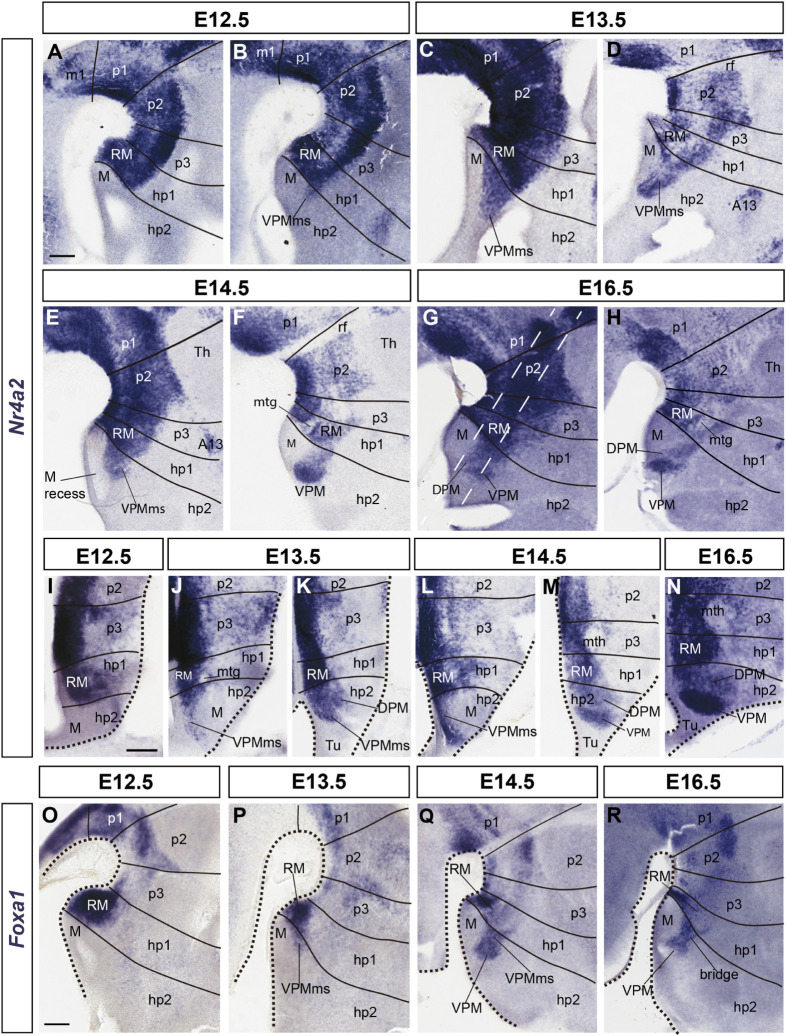
Details of developmental progress of the VPMms and VPM nucleus. **(A–H)** Sagittal *Nr4a2* ISH sections between E12.5 and E16.5. **(I–N)** Horizontal *Nr4a2* ISH sections between E12.5 and E16.5. **(O–R)** Sagittal *Foxa1* ISH sections between E12.5 and E16.5. Black lines mark hypothalamic, diencephalic, and rostral mesencephalic interprosomeric limits (units hp2, hp1, p3, p2, p1, m1), perpendicular to longitudinal references, here to the floor plate (compare Figure 1C). In **(A–H)** each embryonic day is represented by two sagittal sections, the first one being medial to the other. The section planes of the horizontal *Nr4a2* sections (particularly **J–M**) are indicated with white dash lines in **(G)**. In duplets of horizontal sections of the same embryonic day, the ventral section crossing the mamillary area **(J,L)** is followed by a more dorsal section through the main VPMms **(K,M)**. **(O–R)** These sagittal sections labeled with *Foxa1* ISH reveal that this marker predominates in the ventral part of RM (compare with *Nr4a2* signal in **A–H**). Scale bars represent 200 μm.

Our preliminary analysis of such expression data suggested selecting the genes *Nr4a2* and *Foxa1* as convenient markers of the predicted VPM migration. Both *Nr4a2* and *Foxa1* show strong expression from early stages onward at the RM area, while the overlying basal region is primarily devoid of such signals (this includes M, PM, and Tu; Figures 1D,F, 2A,B, 3A,B,O). A sequential follow-up across successive stages provides descriptive evidence for the migrating VPM (e.g., Figures 2C–F, 3C–N,P–R). Interestingly, *Nr4a2* labels selectively the rostrally directed VPM cell population, but not the RM cells entering dorsalward the subthalamic subpial migrating population (Gilbert, 1935; Keyser, 1972, 1979; Altman and Bayer, 1978; Marchand, 1987; Jiao et al., 2000; Martin et al., 2002, 2004; Skidmore et al., 2008). *Foxa1* labels instead both migration streams (Figures 2I,J). We thus identified a distinct VPM migration stream (VPMms).

To follow the essential steps in the development of the VPM nucleus, we will illustrate images of mouse embryos between E12.5 and E18.5, mapping by double immunofluorescence or ISH the *Foxa1* and *Nr4a2* patterns observed in the ventrobasal hypothalamic area.

The mamillary primordium is clearly negative for both markers at E12.5/E13.5 (M; Figures 1D,E, 2A–D, 3A–D,I,J,O,P). *Nr4a2* appears expressed strongly in the mes-diencephalic basal plate (where it relates to developing dopaminergic cell populations) and the hypothalamic RM (RM; Figures 2A–D, 3A–D,I–K). The VPM primordium cannot be identified at E12.5/E13.5, though the VPMms is incipiently present in *Nr4a2* material at E12.5, and it starts to cross the PM domain at E13.5 (VPMms; Figures 1D[inset],E, 3B–D,J,K, 4A,A’).

**FIGURE 4:**
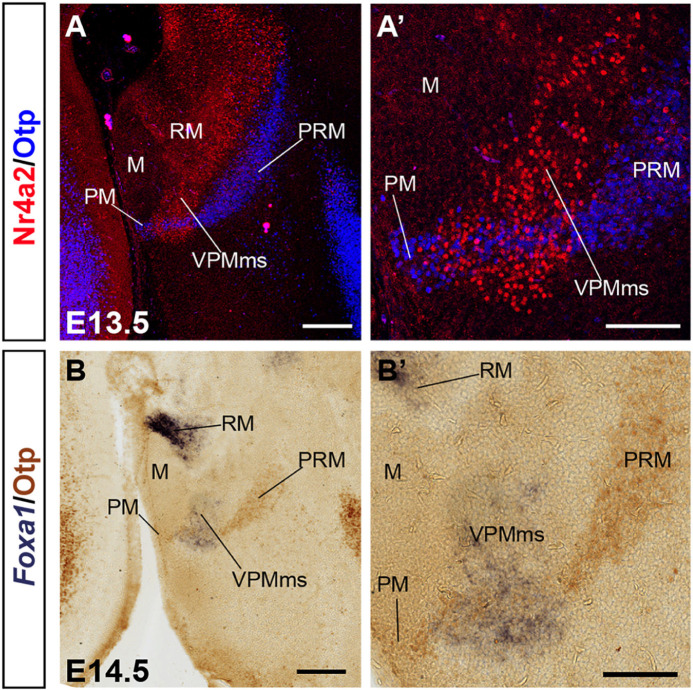
VPMms crosses obliquely the PRM-PM band at E13.5. **(A,A’)** Sagittal section at E13.5 and higher magnification detail showing double immunofluorescence for Nr4a2 (red) and Otp (blue) illustrating Nr4a2-positive VPMms bridge cells across the PM band. **(B,B’)** Sagittal section at E14.5 and higher magnification detail illustrating *Foxa1 in situ* hybridization and immunohistochemistry for Otp. Migrated *Foxa1*-positive cells of the VPMms bridge lie across the PM band. Scale bars in **(A,B)** represent 200 μm. Scale bars in **(A’,B’)** represent 100 μm.

Though *Nr4a2* and *Foxa1* show a partially overlapping pattern, there are some differences in their distribution within RM at E12.5/E13.5. *Foxa1* is transcribed in the ventricular zone, whereas *Nr4a2* is absent in that stratum, being restricted to the mantle. Moreover, *Foxa1* is more widely expressed in the ventral part of RM, whereas *Nr4a2* expression is more abundant in the dorsal part (RM; Figures 2A–D, 3A–D,O,P). Nevertheless, there is an intermediate RM subarea where Foxa1 and Nr4a2 immunoreaction signals co-localize (yellow signal in Figures 2C,E; arrowheads). This result suggests the existence of three cell types in relation to the analyzed markers (green-fluorescent cells express only Foxa1, magenta-fluorescent cells express Nr4a2, and yellow cells apparently co-express Foxa1/Nr4a2). As mentioned above, the RM and M areas are delimited dorsally by a molecularly distinct longitudinal band previously defined as the perimamillary/periretromamillary progenitor area across the basal hypothalamus (PRM/PM; Figures 1C,E,G,H, 4; Puelles et al., 2012). This band selectively expresses *Otp* and *Sim1*. Puelles et al. (2012) ascribed to the PM domain the origin and differentiation of the classic dorsal premamillary nucleus (DPM; Figures 3G,H,K,M,N). We will see that our object of interest, the VPMms, crosses obliquely the PM part of this band (VPMms; Figures 1E, 4). Corroborating the pioneering observations of Puelles et al. (2012), the PM gradually becomes crossed by a stream of Nr4a2-immunoreactive and/or *Foxa1-*expressing RM cells over the E13.5-E14.5 period (Figure 4).

Ventrally to the VPMms there appears distinctly also at E13.5 an incipient *Nr4a2*-expressing DPM stream, which diverges from the VPMms as soon as the cells enter the tuberal area (DPM; Figure 3K). This result suggests that both the DPM and VPM cell groups, or at least some of their subpopulations, grow out from the RM. The two streams become mutually distinct as they invade the PM band, or, passing beyond the PM, reach the tuberal area, respectively.

At E14.5, the RM area appears distinctly divided into a field of green-fluorescent Foxa1 cells, more abundant ventrally and medially, and a partly overlapping field of magenta-fluorescent Nr4a2 cells, which predominate dorsally and laterally. These RM subdomains are connected by a narrow band of yellow double-fluorescent cells which apparently co-express these two markers (RM; arrowheads in Figure 2E). At this stage of development, both green-fluorescent (Foxa1) and magenta-fluorescent cell (Nr4a2) populations extend obliquely rostralward across the perimamillary band, entering the VPMms. The latter reaches with its tip the ventral intermediate tuberal area, where the rounded VPM primordium starts to emerge as a mixture of Foxa1 and Nr4a2-positive cells, with some double-labeled cells (VPM; Figures 2E,F,F’,F”,F”’,F””, 3E,F,L,M,Q, 4B,B’). The nucleus aggregates in a slightly more lateral position within the ventral tuberal intermediate area, since the VPMms diverges lateralwards as it encounters the local acroterminal domain (infundibulum and rostromedian tuberomamillary area). The emergent VPM nucleus soon acquires an ovoidal or rounded shape, with a tip pointing to the lateral pial surface (VPM; Figures 3E,F). The underlying, selectively Nr4a2-positive, DPM primordium, that forms within the perimamillary band, shows stronger labeling intensity than previously (DPM; Figure 3M). Details of the distribution of magenta Nr4a2 cells and green Foxa1 cells within VPM reveal that the Foxa1-Nr4a2 population predominates inside VPM and seems less connected with the trailing VPMms (Figures 2F’,F”,F”’,F””), while the green cells largely overlap the red VPM cells, but show a denser connection with the VPMms (Figures 2F”,F”’). At E16.5 the VPMms has diminished in size, as both DPM and VPM have reached their definitive positions within PM and TuV-TuI, respectively (Figures 3G,H,N,R).

At E18.5 we still observe a non-homogeneous distribution of Foxa1 versus Nr4a2 cells inside the RM territory. Foxa1 cells are densely grouped ventrally, while Nr4a2 cells mainly aggregate dorsally (Figure 2G), defining the dorsal border of RM. In lateral sagittal sections, as already observed from E13.5 onward, the RM area shows a dorsal subpial extension of the Foxa1-positive RM population into the well-known subthalamic/parasubthalamic migration stream, that is restricted in its course to basal retrotuberal PHy. This stream is wholly separated from the rostrally directed VPMms. The Nr4a2-positive RM cell population does not participate in the subthalamic/parasubthalamic migration (STh; PSTh; Figures 2I,J).

A non-homogeneous distribution of cell types within VPM was manifest in horizontal sections at E18.5. The Foxa1 population predominates particularly at the more superficial lateral part of the VPM nucleus (VPMlat; Figures 2I,I’,J,J’). Both sorts of cells, as well as the double-labeled Foxa1-Nr4a2 cells, populate densely the central part or core of the VPM, which encloses the majority of Nr4a2-labeled cells (VPMc; yellow and magenta-fluorescent cells; Figures 2H,J,H’,J’). The remnant of the VPMms that connects periventricularly VPM with RM is also composed mainly by Foxa1 cells (green-fluorescent cells; arrowheads; Figure 2G). VPM Nr4a2 cells seem slightly less connected than Foxa1 cells with RM along the bridge remnant of the VPMms, also visible belatedly with Irs4 (Figures 2G,K).

### Comparison With Other Gene Markers

Immunohistochemical and ISH data were collected from E18.5 embryos cut sagittally, to check the general disposition of *Foxa1* and *Nr4a2* cells with neighboring ventro-basal hypothalamic structures. We first compared with molecular markers expressed near the RM, VPMms, and VPM. In a second step, we examined additional genes that label cell migrations coming out of the RM area, including those coursing into the subthalamic complex.

**FIGURE 5:**
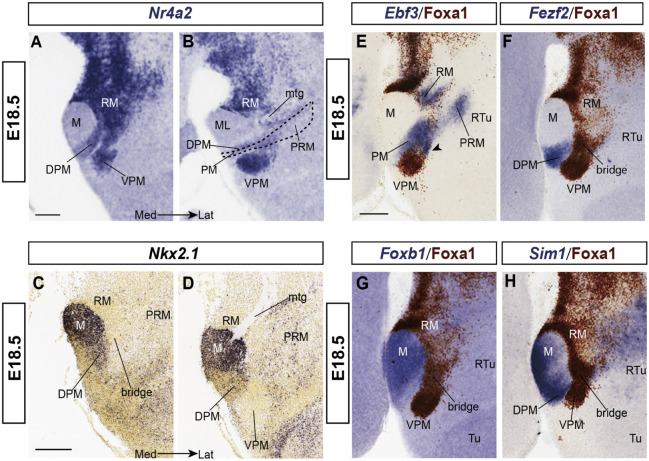
Labeling of some neighboring structures next to RM and VPM at E18.5. **(A,B)** Two medio-lateral sagittal sections showing the DPM ventral to VPM; this smaller population also seems to migrate out of RM **(A)** into the PM band (dash line in **B**); it is also labeled with *Nr4a2* ISH. **(C,D)** Two medio-lateral sagittal sections from the Allen Developing Mouse Brain Atlas labeled with *Nkx2.1* ISH, a widespread basal plate marker in the hypothalamus *except* at the RM area **(C,D)**, showing the likewise negative VPMms bridge **(C)** and VPM **(D)** areas. **(E–H)** *Ebf3, Fezf2, Foxb1*, and *Sim1* ISH combined with Foxa1 immunoreaction to show mamillary markers (M in **G,H**), perimamillary/periretromamillary labeling (PM/PRM in **E**), and DPM signal (in **F,H**) next to Foxa1.positive RM, VPMms and VPM. Scale bars represents 200 μm.

*Nr4a2* reveals VPM and DPM at E18.5 (Figures 5A,B) whereas *Nkx2.1* signal labels the mantle of the entire hypothalamic basal plate, with exception of periretromamillary, RM, VPMms and VPM (Figures 5C,D). Remarkably, PM mainly expresses *Nkx2.1* at its caudal and rostral ends (PM; Figures 5C,D), whereas the PM portion occupied by the migrated DPM population shows very little *Nkx2.1* signal (DPM; Figures 5C,D; compare Figures 5A,B).

*Ebf3* appears from E13.5 onward at the PM area, as well as in a part of RM and the periretromamillary area (RM; PRM; Figure 5E). Where the PRM/PM band is crossed by the VPMms, some *Ebf3*-positive cells seem to deviate from PM into the bridge remnant of VPMms (arrowhead; Figure 5E). *Fezf2* appears expressed also within DPM (Figure 5F).

**FIGURE 6:**
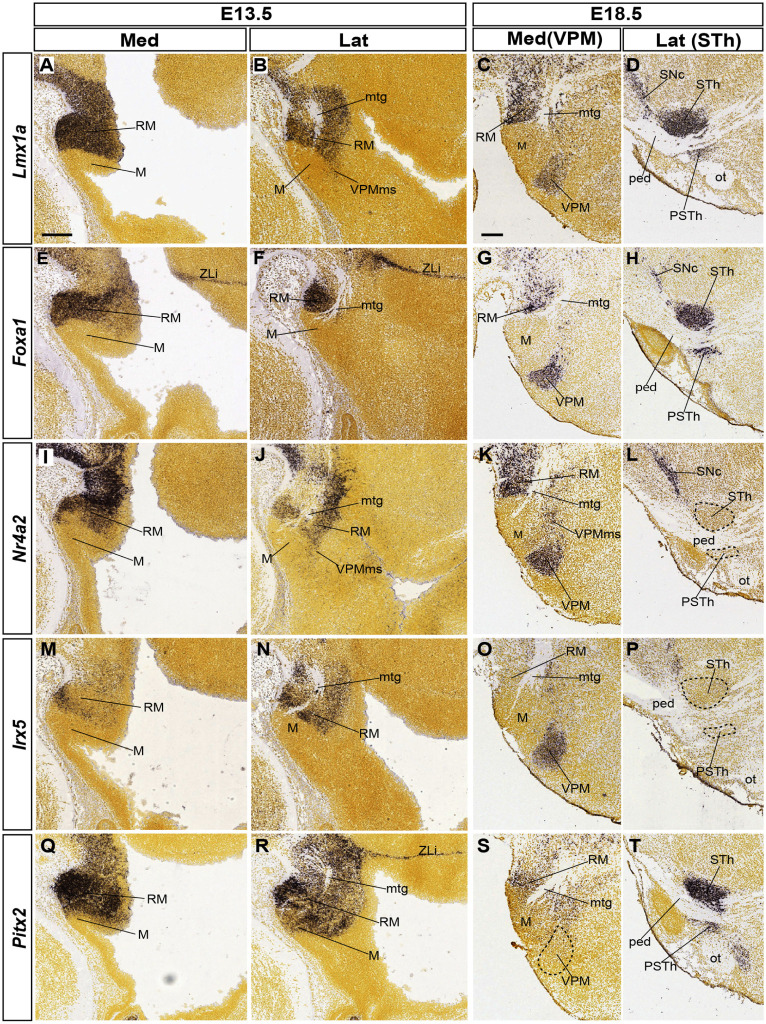
Various retromamillary markers and their differential involvement in tangential migrations. The five indicated gene markers are expressed at least partially in the RM area at E13.5 (medio-lateral sagittal sections in **A,B,E,F,I,J, M,N, Q,R**; sagittal images from the Allen Developing Mouse Brain Atlas). Comparison with equivalent medio-lateral sections at E18.5 **(C,D,G,H, K,L,O,P,S,T)** shows three sorts of results regarding the possible labeling of either VPM or STh/PSTh populations migrated out of the RM area: *Lmx1a* and *Foxa1* label both derivatives (VPM, STh/PSTh in **C,D,G,H**); *Nr42* or *Irx5* label only VPM **(K,L,O,P)**; *Pitx2* labels only STh/PSTh **(S,T)**. Scale bars represent 200 μm.

*Foxb1* is a selective marker for the M area (Figure 5G), and *Sim1* labels both the mamillary area plus the PRM/PM domain (Figure 5H; see the PRM signal in the Allen Developing Mouse Brain Atlas). These two markers are absent from RM, VPMms and VPM. In contrast, *Nr4a2* and *Foxa1* (Figures 5A,B,E–H), and also, *Irx5* and *Lmx1a* (see Figure 6) are positive at RM, VPMms and VPM, but negative in the M, PM, and PRM areas.

*Lmx1a* appears strongly expressed at E13.5 in the whole RM area (Figures 6A,B). Medial sections show expression at the RM ventricular zone, possibly at both floor and basal plate levels (Figure 6A). The incipient VPMms appears labeled in lateral sections through the mantle zone (Figure 6B). At E18.5 there remains label at the RM, and both migrated VPM and STh nuclei appear labeled separately at different mediolateral levels of section (Figures 6C,D). The parasubthalamic nucleus, also labeled by *Lmx1a*, is another RM derivative that migrates less compactly dorsalward into a more rostral part of retrotuberal area (Figure 6D).

The *Foxa1* labeling pattern is roughly comparable to that of *Lmx1a* (RM, VPM, STh, PSTh; Figures 6E–H). Transcripts characterize also both the local basal and floor ventricular zone (Figure 6E and observations not shown); nevertheless, the *Foxa1*-positive mantle tends to aggregate ventrally (Figure 6E), and is seen laterally only under the mamillotegmental tract (Mtg), possibly leading into the STh migration stream (mtg; Figures 6F–H). In contrast, *Nr4a2* signal is extensive at the floor and basal parts of RM at E13.5, whereas only scarce floor *Irx5* signal appears at the rostralmost RM (Figures 6I,M). In any case, both markers appear laterally throughout the RM mantle, surrounding ventrally and dorsally the Mtg (Figures 6J,N). Both markers also distinctly label out of the dorsal RM mantle the E18.5 VPM and its connecting bridge, but the STh or PSTh cell populations remain unlabeled (VPM; STh; Figures 6K,L,O,P). Finally, *Pitx2* transcripts are abundantly present at the E13.5 RM area (Figures 6Q,R), as well as the E18.5 STh and PSTh nuclei, including mainly ventrally placed RM remnants (RM, STh, PSTh; Figures 6S,T). This marker is clearly absent at the VPM locus (Figure 6S, compare with Figures 6G,K,O).

### Experimental Tracing of the VPM Migration

**FIGURE 7:**
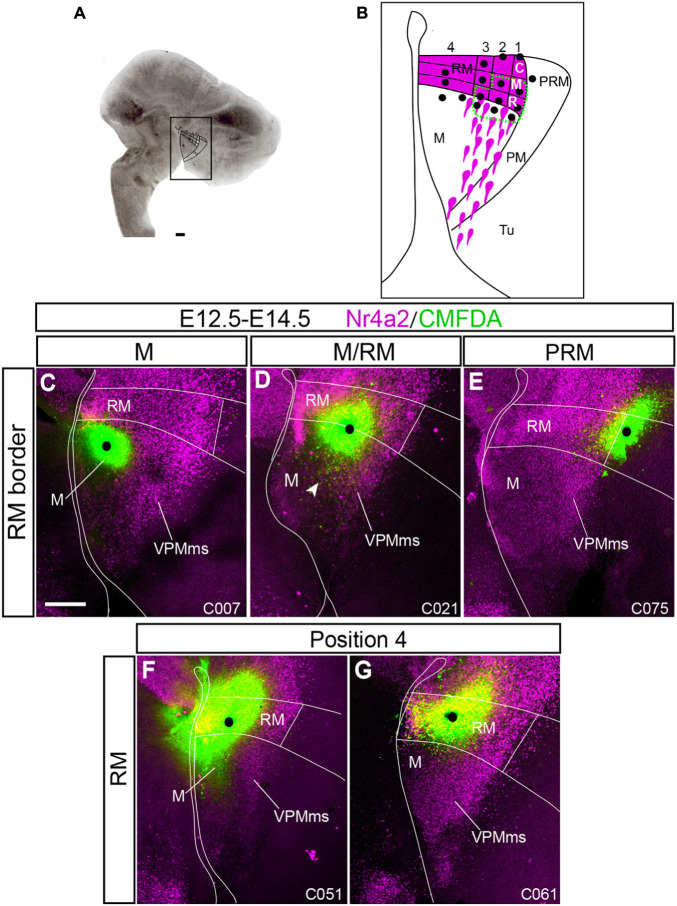
Control experiments showing no VPMms. To narrow the optimal labeling area for VPMms within RM we marked several points inside and outside RM in our organotypic cultures (see section “Materials and Methods”) using an *ad hoc* subdivision system. CMDFA signal is in green and Nr4a2 immunofluorescence is in magenta. The alphanumeric identification of the cases appears at the lower right corner (compare Supplementary Table 3). **(A)** Example of our organotypic culture material in which RM, M, PRM, and PM areas are marked. **(B)** Schema of our arbitrary RM subdivision system. We divided RM (magenta) into dorsoventral halves, and the upper half in three longitudinal tiers, subdivided into three rostro-caudal sectors (rostral, middle, caudal). Black dots represent the marking sites of experimental cases, some of which are shown in Figures 7, 8. Experiments outside the area limited with a green dashed line showed no VPMms (Figure 7), whereas those inside it resulted in distinct VPMms labeling (stream of green cells; Figure 8). **(C)** No migratory stream was observed when a CMFDA mark was placed strictly within the mamillary area (M; *n* = 5). **(D)** CMFDA-tungsten grains placed just in front of the M/RM boundary in an area traversed by some migrating cells showed only minor displacements toward the M area (arrowhead in **D;** *n* = 10). **(E)** CMFDA grains placed in PRM, even close to RM, did not elicit VPMms labeling (*n* = 5). **(F,G)** Experiments labeling the lower half of RM (position 4 in the schema in **B**) did not show marking of VPMms (*n* = 3). Scale bars represent 200 μm.

**FIGURE 8:**
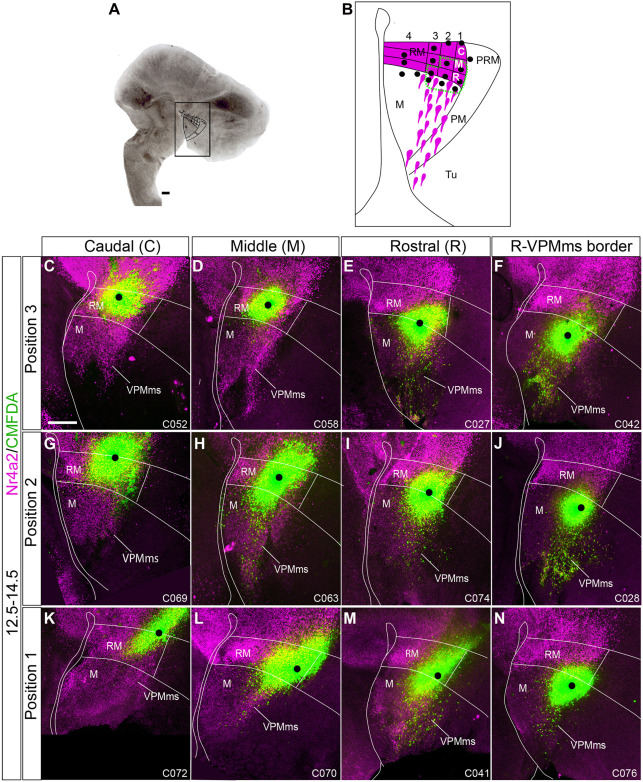
Origin of VPMms in the dorsal half of RM. **(A,B)** We visualize here representative cases with marking positions within the upper half of RM, part of which labeled the VPMms; some cases labeling the VPMms itself rostral to RM are shown as well; we employ the same reference system used in Figure 7 (dorsoventral positions 1–3 indicated at the left, and rostrocaudal locations C/M/R above). CMDFA marking signal is in green and Nr4a2 immunofluorescence is in magenta. The black dots indicate the observed labeling grain; we estimate that migrating cells lying mainly close to the grain were sufficiently labeled, irrespective of accompanying diffusion of label around it. The alphanumeric identification of the cases appears at the lower right corner (compare Supplementary Table 1). All cases marked in the C or M domains of position 3 (ventral tier of upper RM half) were negative (**C,D**; 3C *n* = 2, 3M *n* = 3), as were the cases marked in the C domain of positions 2 or 1 (**G,K**; 2C *n* = 1, 1C *n* = 1). The remaining cases shown were positive and show a distinct rostralward dispersion of fluorescent labeled cells (VPMms), each experiment visibly identifying only a fraction of the total VPMms cell population visualized with background Nr4a2 labeling (**E,F,H–J,L–N**; 3R *n* = 12; 3VPMms *n* = 12; 2M *n* = 3; 2R *n* = 6; 2VPMms *n* = 8; 1M *n* = 3; 1R *n* = 2; 1VPMms *n* = 3 Supplementary Table 3). Scale bars represent 200 μm.

We performed organotypic *in vitro* experiments on E11.5, E12.5, and E13.5 mouse embryo half-brain explants in order to trace the advance of the VPM migration after application of a small grain of CMFDA fluorescent tracer at chosen places of the explant’s ventricular surface (see section “Materials and Methods”). All labeled explants were maintained 48 h in incubation under 5% CO2 and 37°C (they were thus named indicating the day of labeling and the day of fixation, e.g., E12.5–E14.5). We investigated the extent and temporal profile of the migration and determined roughly the apparent origin of the VPM migratory stream within the RM area. To this last aim, we mapped the different experiments on a standard set of arbitrary dorsoventral and rostrocaudal subdivisions of the RM area (see section “Materials and Methods”). This helped us to assess the success obtained in labeling the migration depending on the relative starting position labeled within RM (or along the VPMms) (Figures 7A,B, 8A,B). Moreover, to precisely visualize the relative position of the labeled cells within the VPMms, we systematically performed immunofluorescent counterstaining with anti-Nr4a2 antibody. Sometimes we added (using double immunofluorescence) anti-Foxa1 antibody (labeling both the VPM and STh migration streams), or an anti-Otp antibody (Otp is a selective marker of the PRM/PM band; Puelles et al., 2012). As control experiments, we also marked similarly with CMFDA fluorescent tracer several other neighboring positions outside of the RM area. These controls included the mamillary body (M; Figure 7C) and the periretromamillary area (PRM; Figure 7E).

We considered that a RM labeling case was positive (indicating tangential migration) whenever we saw labeled cells outside the RM area migrating toward the tuberal domain.

**FIGURE 9:**
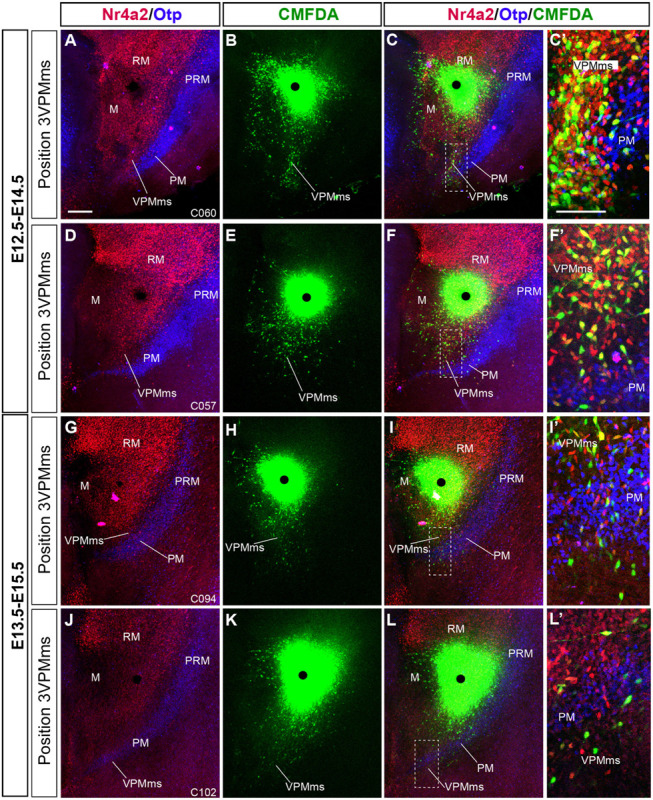
Comparison of VPMms labeling experiments performed at E12.5 and E13.5. We illustrate similarly as in Figure 11, with Nr4a2 and Otp background labeling of RM/VPMms and PRM/PM, respectively, two representative cases of our E12.5–14.5 series **(A–F)**, compared with two cases of our E13.5–E15.5 series **(G–L)**. The number of labeled VPMms cells seems to decrease in the latter group. **(C’,F’,I’,L’)** Higher magnification images taken from the boxed-in areas in **(C,F,I,L)**, showing fluorescence-typological VPMms cell details, as observed in Figure 11. Note that E12.5-E14.5 cases have more CMFDA + Nr4a2 (yellow) cells than E13.5–E15.5 cases. Scale bar in **(A)** represents 200 μm valid for **(B–L)**. Scale bar in **(C’)** represents 100 μm and is valid for **(F’–L’)**.

We will first comment on some general aspects relative to the experimental results obtained. Importantly, as regards timing, we had no positive case among E11.5–E13.5 experiments, and most of the positive cases were obtained in E12.5–E14.5 experiments (Supplementary Table 3). Most E13.5–E15.5 cases were positive, but showed little migration in terms of distance covered, perhaps indicating a terminal slowing down of the migration (Supplementary Table 4 and Figure 9). The effective labeling points were deduced from the relative positions of the inserted CMFDA grains, according to our arbitrary map of RM subdivisions superposed on the cultured tissue, irrespective of the spread of labeling around it. We marked with a black dot the locus where the labeling grain was in our photographs of such experiments. Interestingly, several experiments labeled a rostro-ventral stream, toward the M area (arrowhead in Figure 7D).

### Precise RM Origin of the Migration

We separately labeled various retromamillary loci (Figures 7, 8); and some other points in the neighboring neuroepithelium, sampling also M and PRM. No evidence of migration toward the overlying tuberal area was found in markings done at M (*n* = 5; Figure 7C), or across the RM/M limit (*n* = 10; Figure 7D). The VPMms also remained unlabeled when the PRM was marked dorsally to RM or bridging the PRM/RTu boundary (*n* = 5; Figure 7E).

As regards RM experiments, referring basically to E12.5–E14.5 (*n* = 79; Supplementary Table 3) experiments, their success depended on a relative rostrodorsal position identified within the *ad hoc* RM map shown in Figures 7B, 8B, defining four dorsoventral positions (1–4), each subdivided into three possible anteroposterior marking sites (C, M, R).

-Position 4 experiments (ventral half of RM): This area represents the narrower ventral zone of RM, where *Foxa1* expression predominates. Remarkably, none of the cases marked at this position labeled the VPMms (*n* = 3; Figures 7F,G).

-Position 3 experiments: This is the ventralmost tier of the upper RM half. No labeled migrating VPMms cells were observed in experiments labeling its caudal part (3C; *n* = 2, Figure 8C) or its middle portion (3M; *n* = 3; Figure 8D). The positive cases obtained in this tier were marked at the 3R site (*n* = 12; Figure 8E), or in experiments slightly rostral to this locus, already touching the VPMms proper (*n* = 12, Figure 8F). The distance traveled by CMFDA-labeled cells originating at the 3R site (or correlative initial VPMms) by E14.5 (2 days survival *in vitro*) roughly corresponded to the VPMms length revealed by Nr4a2 immunoreaction.

-Position 2 experiments: This corresponds to the intermediate tier of the upper half of RM. In this tier, cases marked at the sites 2M (*n* = 3; Figure 8H), 2R (*n* = 6; Figure 8I), and the correlative start of the VPMms (*n* = 8; Figure 8J) showed labeled cells along the VPMms while cases marking the 2C site (*n* = 1; Figure 8G) were unsuccessful. These positions along tier 2 roughly lie at the transition between ventral *Foxa1* expression and dorsal *Nr4a2* signal in RM. The 2M experiments were the caudalmost ones showing significant CMFDA labeling of the VPMms.

-Position 1 experiments: The first tier of RM lies dorsalmost, just ventral to the *Otp*-positive PRM band. As in the other dorsal RM tiers, markings at the 1C site did not label the VPMms (*n* = 1; Figure 8K). The cases that labeled the 1M site (*n* = 3; Figure 8L) labeled migrated cells only up to the proximal part of VPMms. The positive cases marked at the 1R site showed instead a significant group of labeled cells distributed practically along the whole length of the VPMms (*n* = 2; Figure 8M), as well as VPMms cases (*n* = 3; Figure 8N).

### Cell Profile in E12.5–E14.5 and E13.5–15.5 Experiments

**FIGURE 10:**
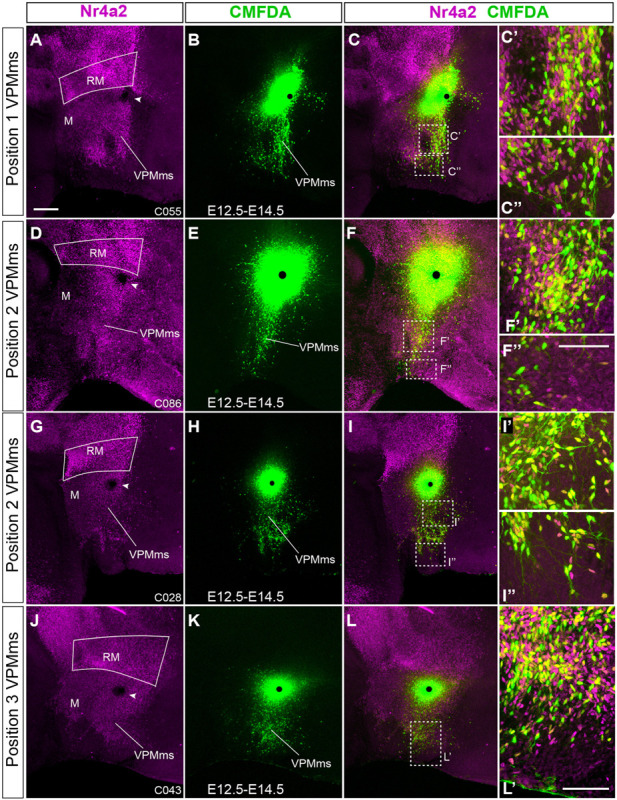
Examples of E12.5-E14.5 cases marked within the VPMms proper. **(A–C,D–F,G–I,J–L)** illustrate each complete case. **(A,D,G,J)** Nr4a2 immunofluorescence in magenta; the arrowhead indicates the labeling site. **(B,E,H,K)** CMFDA signal corresponding to each case; the black dots indicate the labeling sites. **(C,F,I,L)** Superposed images of the two channels (Nr4a2 and CMFDA). **(C’,C”, F’,F”, I’,I”,L’)** Higher magnification details of immunofluorescent typology of the marked migrated cells taken at the sites boxed-in in **(C,F,I,L)**. Yellow cells co-localize both Nr4a2 and CMFDA signals, indicating migrated Nr4a2 cells. Magenta cells are also migrated Nr4a2 cells which were not labeled in this experiment. Green cells are labeled migrated cells that do not express Nr4a2 (i.e., they express Foxa1 or another marker). Note we do not see the theoretically present unlabeled cells lacking Nr4a2 signal. Scale bar in **(A)** represents 200 μm, valid for **(D**,**G**,**J)**. Scale bar in **(F”)** represents 100 μm being the same for **(C’,C”, F’,I’,I”)**. Scale bar in **(L’)** represents 100 μm.

We show in Figures 10A–L four E12.5–E14.5 cases showing maximal labeling of the VPMms; all were marked at the VPMms itself, at different distances from the rostral RM boundary. We include high magnification details of these migrations (Figures 10C’,C”,F’,F”,I’,I”,L’). These show green-fluorescent cells representing CMFDA-labeled elements (as in the corresponding low magnification images), jointly with red-fluorescent Nr4a2-immunoreactive neurons not labeled with CMFDA, and yellow-fluorescent double-labeled CMFDA/Nr4a2 cells. Note the green fluorescing cells lack Nr4a2 signal; they may represent Foxa1-positive elements, or other components of the VPMms population with an unknown molecular profile.

Given that there are differences in the detailed distribution of Foxa1 and Nr4a2 transcripts within the RM, including at the restricted rostrodorsal RM area where the VPMms apparently originates, we examined the relative distribution of these signals in the VPMms and the VPM proper. To this end, we carried out comparable E12.5–E14.5 labeling experiments, doubly counterstained with Foxa1 and Nr4a2 immunofluorescent reactions (blue versus red signal, respectively; *n* = 4).

**FIGURE 11:**
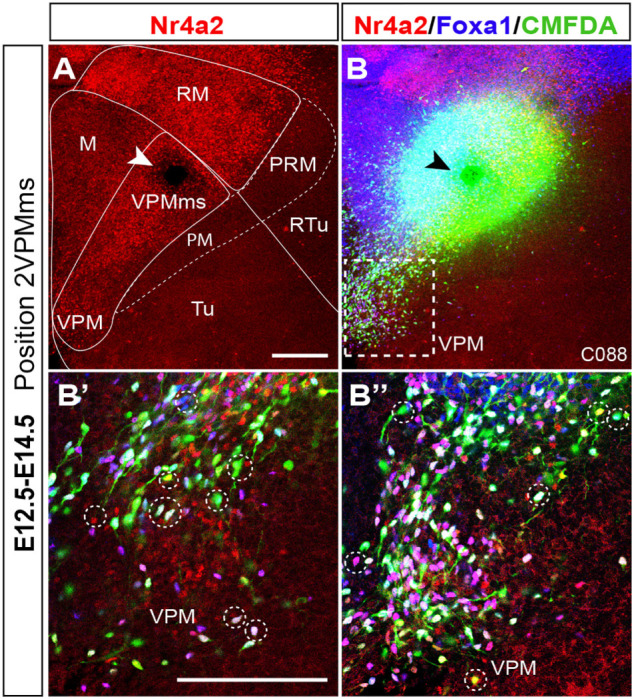
Analysis of VPMms cell molecular diversity. **(A)** Nr4a2 immunoreacted E12.5–E14.5 case marked with CMFDA at the initial part of VPMms (arrowhead indicating grain site). Relevant areal boundaries and areas are indicated. **(B)** Same case showing green fluorescent CMFDA labeling of the VPMms, as well as blue Foxa1 immunoreaction. **(B’,B”)** Higher magnification medial and lateral 3 μm-thick confocal optic sections from **(B)** taken from the boxed-in area in **(B)**. The diverse possible combinations of markers obtained in individual VPMms neurons result in different fluorescent colors sampled in both images by discontinuous circles; Nr4a2 + CMFDA (yellow), Foxa1 + CMFDA (light blue), Nr4a2 + Foxa1 + CMFDA (white), CMFDA + other RM gene markers not analyzed (green). Scale bars represent 200 μm.

We show a representative example of these experiments in Figure 11. The VPMms was labeled at its beginning, just outside the RM, before it starts to cross the PM band (arrowhead; Figures 11A,B). At low magnification Foxa1 cells predominate abundantly at the ventral part of the VPMms, whereas Nr4a2 cells do so dorsally, with some overlap (Figures 11A,B). Scattered Nr4a2 and Foxa1 neurons are visible along the advancing VPMms, reaching the VPM. We also show higher magnification details of the area boxed in Figure 11B, illustrating two individual 3 μm-thick confocal optical slices taken at medial and lateral levels through VPM (Figures 11B’,B”). All cells that were labeled with CMFDA as they moved past the labeling site display green fluorescence, which may combine or not with blue Foxa1 signal or with red Nr4a2 signal. Green plus blue gives a pale blue fluorescent image, which is indeed detected in some cells. Green with red gives a yellow fluorescent signal, also present in the image shown. This indicates that the experiment separately labeled cells of both Foxa1 and Nr4a2 types, as expected. These double-labeled cells appear mixed along the VPMms and VPM with single-labeled cells standing out by pure dark blue or red fluorescence (cells unlabeled with CMFDA). Interestingly, the fact that we also find ‘only green fluorescent’ cells suggests that there exist migrating VPM neurons which do not express either Foxa1 (blue signal) or Nr4a2 (red signal), indicating there is additional uncharacterized molecular heterogeneity within this nucleus and its migration (we are missing one or more markers of the migration). Finally, we observed also white-fluorescent cells, interpreted by us as CMFDA-labeled neurons coexpressing Foxa1 and Nr4a2. White-fluorescent cells seem to be more abundant overall than yellow or greenish-blue cells, though the proportion varies across the different experiments (*n* = 4). Given the availability of only few such cases, and the variability involved in the subtle positional changes in the labeling site, we thought that the sample was not appropriate to perform quantitative analysis.

In Figure 9 we show four similar double counterstained cases, comparing E12.5–E14.5 (*n* = 79; Supplementary Table 1) and E13.5–E15.5 (*n* = 17; Supplementary Table 4) results. The E12.5–E14.5 experiments show long migrations of the VPMms cells, which start to cross the blue-fluorescent Otp-labeled PM band (Figures 9A–F). The E13.5–E15.5 cases show instead only limited migratory advance of labeled cells (Figures 9G–L). In both cases aberrant cells are observed that enter the mamillary area (M). The higher magnification (Figures 9C’,F’,I’,L’) details shown at the right for every case illustrate the variety of fluorescence reactions observed (discussed in the previous paragraph).

### Altered Phenotype of the VPMms and VPM in Fgf8 Hypomorphs

**FIGURE 12:**
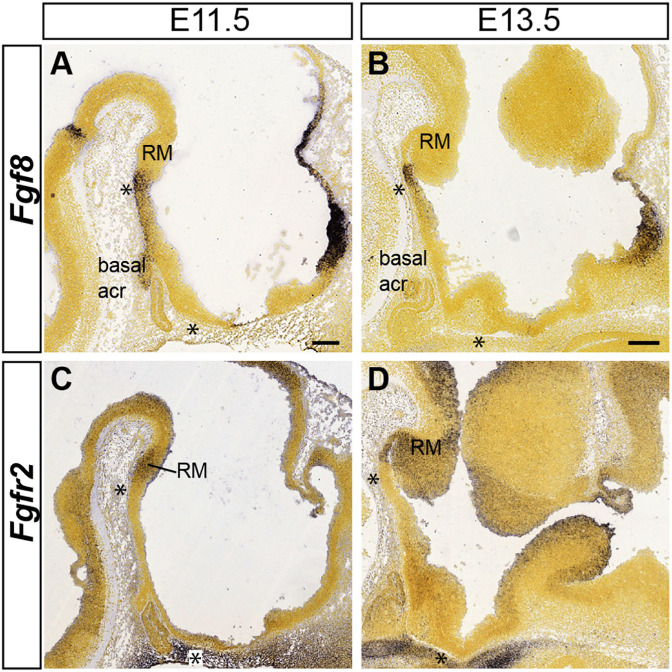
Mamillary acroterminal expression of *Fgf8* and RM signal of its receptor *Fgfr2* at early stages. Allen Developing Mouse Brain Atlas images are shown to illustrate the close location of an acroterminal FGF8 source to one of its possible receptors at the RM. **(A)** *Fgf8* is active at the mamillary part of the acroterminal basal plate at E11.5. **(B)** At E13.5 *Fgf8* expression becomes restricted at the ventralmost mamillary acroterminal domain (see asterisks in **A**,**B**). **(C,D)** The diffusible FGF8 protein (out of the asterisk-marked acroterminal domain) can activate transcripts of the Fgfr2 receptor produced in the hypothalamic basal plate, including RM at both E11.5 and E13.5. Scale bar in **(A)** represents 200 μm and is valid for **(C)**. Scale bar in **(B)** represents 200 μm and is valid for **(D)**.

In our search of possible causal mechanisms related to the migration of the VPM, one line investigated was the possibility of an attracting signal rostral to the RM, which might trigger and guide the VPM migration. The immediate rostral neighbor of the RM area, the mamillary area, was not a candidate as an attractor source, because the migration largely evades entering that domain; it rather seems that M is a non-permissive domain for the advance of the VPMms. We know of no salient cell population which might release attracting molecules at the tuberal site targeted by the VPM cells. However, the VPM cells stop their migration just as they reach the rostromedian acroterminal hypothalamic domain. The prosomeric model postulates there is a molecularly distinct basal acroterminal domain rostral to the M (Puelles et al., 2012; Puelles and Rubenstein, 2015; Ferran et al., 2015). This rostromedian neuroepithelial locus may serve as a secondary organizer, due to its expression of *Fgf* family genes (Ferran et al., 2015; Puelles, 2017; Diaz and Puelles, 2020). We show in Figures 12A,B Allen Developing Mouse Brain Atlas images illustrating mouse acroterminal *Fgf8* transcripts at E11.5 and E13.5, encompassing the period in which the VPM migration occurs. The *Fgf8* expression clearly stops short of the developing mamillary body. In addition, we consulted the distribution of Fgfs receptors (Fgfr1-4) at the Allen Developing Mouse Brain Atlas. The gene coding *Fgfr2*, a prototypic Fgf8 receptor, is expressed at the RM floor and basal plates at E11.5 and E13.5 (Figures 12C,D). We had access to severe Fgf8 hypomorphs (Fgf8^neo/null^ transgenic mice, *n* = 2), in which the release of Fgf8 protein is curtailed.

**FIGURE 13:**
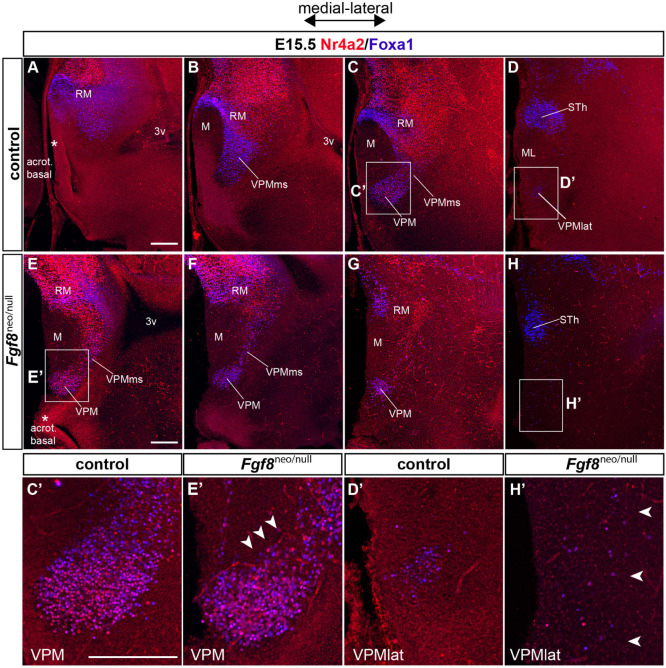
Phenotype of Fgf8 severe hypomorph mice at E15.5. **(A–D)** Medio-lateral sagittal 50 μm-thick sections of an E15.5 control embryo labeled with Foxa1 (blue) and Nr4a2 (red). **(E–H)** Medio-lateral sagittal 50 μm-thick sections of a hypomorph Fgf8^neo/null^ E15.5 embryo labeled likewise with double Foxa1/Nr4a2 immunofluorescence. Note the hypomorph VPMms/VPM sections in **(E–G)** roughly correspond to wildtype VPM sections in **(C)**, suggesting that part of the hypomorph VPMms/VPM lies at abnormally medial positions. The lateralmost VPM cell population disappears earlier in the Fgf8^neo/null^ specimen compared with the wildtype. Moreover, the Foxa1-positive subthalamic nucleus (STh) is distinctly smaller in the hypomorph than in the wildtype specimen (compare STh in **H** versus **D**). **(C’,D’,E’,H’)** Higher magnification comparison of wildtype and hypomorph pairs of images taken from the boxed-in areas in **(C,D,E,H)**. **(E’)** Apparently, the ventral part of VPM shows relatively less Foxa1 cells in the mutant (arrowheads). **(H’)** At lateral levels of the Fgf8^neo/null^ specimen corresponding to the lateralmost VPM cells in the wildtype (arrowheads; **H**), few Foxa1-Nr4a2 positive cells appear abnormally scattered at the expected VPM locus (arrowheads). Scale bars represent 200 μm.

At first, we compared RM and VPM structures by double Foxa1/Nr4a2 immunofluorescence in wildtype and Fgf8^neo/null^ specimens at E15.5. The VPM nucleus appears closer to the midline in Fgf8^neo/null^ mice (Figures 13E–H) than in the wildtype (Figures 13A–D), suggesting a partial atrophy of the whole basal hypothalamus. In the mutant phenotype, the structure of the RM area has a disorganized aspect at both medial and lateral levels, regarding Foxa1 and Nr4a2 markers. There is also a notable decrease of Foxa1-positive RM cells in Fgf8^neo/null^ mice (RM; Figures 13E–G compared with Figures 13A–C). In line with this, the Foxa1-positive population normally seen at the ventral part of the VPM is absent (white arrowheads in Figure 13E’ compared with Figure 13C’). The lateral RM subarea is diminished in the Fgf8^neo/null^ mice. In addition, disperse cells are found in the place where the VPM is found laterally in wildtype mice (Figure 13H,H’ compared with Figures 13D,D’).

**FIGURE 14:**
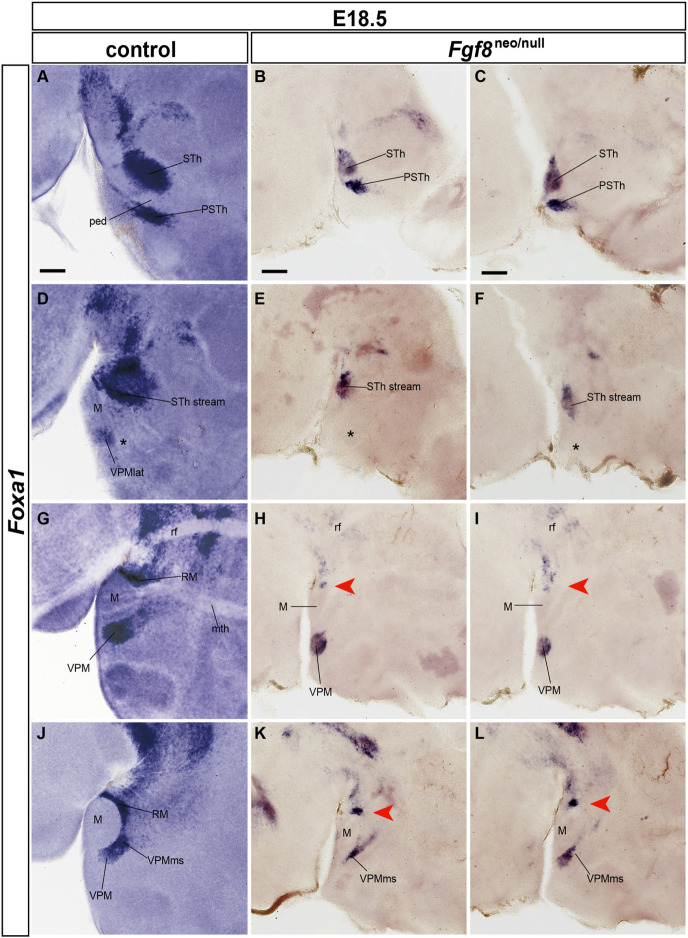
Severe Fgf8^neo/null^ hypomorphs display RM, VPMms, VPM and STh atrophy at E18.5. **(A,D,G,J)** Latero-medially ordered sagittal sections of a wildtype E18.5 embryo labeled with *Foxa1* ISH to show RM, STh and VPM cell populations. **(B,E,H,K)** Right side latero-medial sagittal sections of an E18.5 Fgf8^neo/null^ specimen reacted for *Foxa1* ISH. **(C,F,I,L)** Equivalent left-side sections of the same E18.5 Fgf8^neo/null^ specimen. There is a general reduction in size of the mutant compared with the wildtype specimen (note the magnification is the same in both cases). The migrated STh and VPM show a reduced size, though the respective migrations out of RM have occurred. Red arrowheads in **(E,F)** suggest lack of lateralmost VPM cells at levels through the STh nucleus, and red arrowheads in **(H,I,K,L)** suggest a significant reduction in the cellularity of the RM area. Scale bars represent 200 μm.

Figure 14 compares Foxa1 ISH reaction in the postmigratory phenotype of E18.5 wildtype and Fgf8^neo/null^ specimens. The hypomorphs showed in general a reduced brain size (about 50% reduction), though overall hypothalamic morphological structure was conserved. This atrophic pattern also appears reflected in the RM-VPMms-VPM set of structures, as well as in the related migration of the STh and PSTh nuclei out of the caudal part of the RM area. Following the series of sagittal section from lateral to medial, we see most laterally the STh and PSTh nuclei, whose size is diminished in the hypomorph, compared to correlative wildtype formations (Figures 14B,C compared with Figure 14A). Note we illustrate separately both halves of the hypomorph brain to show the same pattern with slight differences. Apart of the visible size difference of these nuclei in single sections, the wildtype has double as many equivalent-thickness sections through these entities as the hypomorph. At the next chosen section level medialwards the hypomorph STh and PSTh populations appear mixed in a single mass at the lateral aspect of the RM area, possibly corresponding to a partially detained subpial subthalamic migratory stream (STh stream; Figures 14E,F compared with Figure 14D). The wildtype series shows at this level a small labeled cell group at the expected lateral position of the VPM, corresponding to the entity we described above as the lateral VPM population (asterisk in Figure 14D). An equivalent cell group is not visible in the hypomorph (asterisks in Figures 14E,F). At the next chosen section level we see both the RM area and the VPM nucleus. Both formations are smaller in the hypomorph (and they also appear in about double as many sections in the wildtype specimen; red arrowheads in Figures 14H,I compared with Figure 14G). The last chosen section level lies medially and shows in the wildtype specimens the larger medial part or core of the VPM, as well as remnants of its migration stream connecting it to the RM area. In the hypomorph the VPM core mass is much reduced in size, as is the trail of labeled cells behind it. Remarkably, the RM area is also severely reduced in size (red arrowheads; Figures 14K,L compared with Figure 14J).

## Discussion

Various evidence supports a RM origin of the hypothalamic VPM neurons, a notion first postulated after descriptive developmental analysis of some gene markers by Puelles et al. (2012). We extended the list of gene markers whose changing expression pattern between E11.5 and E18.5 strongly suggests a rostralward tangential migration of prospective VPM cells out of the RM neighborhood (Supplementary Table 2). Moreover, to test this notion, we performed fluorescent labeling experiments upon organotypic cultures of brain halves explanted at E11.5, E12.5, and E13.5. These explored the possibility of a circumscript area of retromamillary origin and details of the migratory and temporal course of the studied phenomenon.

The RM area originates not only the periventricularly migrating VPM population, but also the likewise tangentially migrating subpial and dorsal stream of STh and PSTh populations (Gilbert, 1935; Keyser, 1972, 1979; Altman and Bayer, 1978; Marchand, 1987; Jiao et al., 2000; Martin et al., 2002, 2004; Skidmore et al., 2008, 2012). Some of our data suggest that the RM also contributes Nr4a2-positive migrating elements (but not Foxa1 expressing cells) to the dorsal premamillary nucleus, which forms within the perimamillary area (Puelles et al., 2012). We identified a primary multiplicity of cell types within the VPM population (at least Foxa1-positive, Nr4a2-positive, and Foxa1/Nr4a2 double positive cells, plus other cells negative for both markers), consistently with a recent single cells transcriptomic analysis of the basal hypothalamus, which identified a diversity of molecular types within the adult VPM (Mickelsen et al., 2020). Finally, we investigated possible signaling mechanisms affecting the VPM migration, and identified an apparent collateral trophic role of FGF8 signal diffusing caudalwards out of the local median acroterminal domain.

The results obtained from our *in vitro* experimental material corroborate straightforwardly the migratory movement of VPM cells into the tuberal area suggested by Puelles et al. (2012), also noted independently by Nouri and Awatramani (2017). This conclusion results from the progressively rostralward changing position of a group of cells expressing molecular markers characteristic of the RM area (Supplementary Table 2) between E11.5 and E15.5. Moreover, some other markers that initially are expressed uniformly in the basal tuberal hypothalamus (the area invaded by the VPM migration), but are not expressed at the RM area, show complementary negative images of the gradual penetration of the terminal tuberal region by negative VPM cells (e.g., *Nkx2.1*, *Zfhx3*, *Ctnnb1*).

In their autoradiographic analysis of rat hypothalamic development, Altman and Bayer (1986) distinguished separate origins for the RM (their supramamillary nucleus) and the mamillary body, but did not relate the VPM to the former. These authors postulated the VPM origin as occurring near its adult location, in the neighborhood of the mamillary recess. They reported relatively early birthdates for the rat VPM between E13 and E15 (interestingly, non-migrating neurons forming the adult medial and lateral RM nuclei have later birthdates between E15 and E17). The VPM tangential migration in the mouse essentially occurs during E12, though it continues to a lesser extent at least during E13. This period agrees with mouse autoradiographic birthdate data obtained by Shimada and Nakamura (1973), establishing the birth of VPM neurons between E11.5 and E14.5. Leptin-responsive neurons present at the VPM show a peak of neurogenesis at E12.5 (Ishii and Bouret, 2012).

Martin et al. (2002, 2004) and Skidmore et al. (2008, 2012) studied the RM domain as a source of *Pitx2*-labeled neurons migrating dorsalwards into the STh (born in the rat also between E13.5 and E15.5, according to Altman and Bayer, 1978). However, VPM cells do not express this marker, thus representing a different neuron type than the subthalamic one, irrespective that *Foxa1* is a shared marker, needed for the subthalamic migration (Gasser et al., 2016). Apparently unnoticed by these authors, some of their images include loss-of-function evidence suggesting that *Foxa1* is also needed for the VPM migration (their Figure 1C and Supplementary Figure 5D). In our descriptive results we combined data of several VPM markers and obtained the impression that there exist 3 or 4 migrating cell types (or more). The notion of diverse molecular types of VPM neurons has been corroborated recently by single-cell transcriptomic analysis of the adult basal hypothalamus, including the VPM (Mickelsen et al., 2020). Most of the VPM markers identified in the Mickelsen et al. (2020) report only start to be expressed after the migration has finished, according to data at the Allen Developing Mouse Brain Atlas (see Supplementary Table 2^1^). Because of their emphasis on late developmental gene markers, these authors did notice the postulated VPM migration (Puelles et al., 2012; Nouri and Awatramani, 2017), nor compared VPM markers with those of the RM area. A convergent migration of molecularly differentially defined neuronal types into a single adult cell nucleus has been described before for the avian and mouse interpeduncular nucleus (Lorente-Cánovas et al., 2012; Moreno-Bravo et al., 2014; Ruiz-Reig et al., 2017; García-Guillén et al., 2020).

The migration seems to take place largely during the 12th day of gestation (few migrating cells detected at E13.5, and none at E11.5). Nevertheless, the VPM nucleus remains connected to its RM origin by a periventricular bridge of similar cells, as was already noted by Puelles et al. (2012; their Figures 8.31, 8.32). After reaching the intermediate tuberal area, the cells forming the tip of the VPM migration stream diverge lateralwards, approaching obliquely the brain surface (best observed in horizontal sections; Figure 2K). This causes that sagittal sections passing through this lateral portion give the impression that the nucleus is disconnected from the RM area. We conjecture that the final lateral divergence of the VPMms may reflect a non-permissive character of the acroterminal domain in front of it.

In their migration, the VPM cells cross obliquely both the Otp-positive perimamillary band and the histidine decarboxylase -positive ventral tuberal band (also known as ‘tuberomamillary area,’ where histaminergic neurons are produced; Puelles et al., 2012). These bands surround dorsally the mamillary body (Shimogori et al., 2010; Puelles et al., 2012). These curved but topologically longitudinal bands are an example of the dorsoventral patterning of the basal hypothalamus postulated in the prosomeric model (RTu/Tu > dorsal to PRM/PM > dorsal to RM/M > dorsal to the floor plate; Figures 1C,G,H; see also following section).

The obliqueness of the VPM migration relative to these two dorsoventrally arranged thin longitudinal domains is an infrequent aspect for which we do not have an explanation. Most known tangential migrations in the brain proceed longitudinally or transversally relative to the local interareal or microzonal boundaries (e.g., inferior olive and pontine rhombic lip migrations; facial motor nucleus and other motoneuronal migrations, interpeduncular migrations, subpallial cells migrating into the pallium). For instance, the subthalamic migration that also emerges subpially out of the RM area proceeds strictly dorsalwards into the retrotuberal area (a transversal course). However, neurons forming some isthmic hindbrain nuclei via tangential migration are known to migrate obliquely (Puelles and Martinez-de-la-Torre, 1987).

A possible explanation of the VPM case is that a strong rostral attractor may override partially an hypothetic initial tendency of VPM cells to migrate dorsalwards (like the companion retromamillary STh and PSTh cells), causing them to proceed instead along the resultant summatory vector. The VPM cells do not advance strictly rostralwards, either, because that would translocate them into the mamillary area (Figures 1C,G,H). The migrating VPM and DPM cells eschew penetrating the mamillary area while advancing rostralwards, with a stronger dorsalward vector noted in VPM than in DPM. A local non-permissive or repellent signal at the M area is also possible (Figures 1C,G,H).

It is likewise unclear why the periventricularly migrating VPM cells finally diverge lateralwards (radially) into the intermediate and superficial strata within the intermediate tuberal area. The migrating stream approaches the local hypothalamic surface before the movement stops, particularly with its *Foxa1*-positive cells (Figures 2J,J’). However, there is apparently nothing there that might attract or force them toward the pia. The median hypothalamic locus lying immediately in front of the migrated VPM is the acroterminal median eminence, which remains totally free of VPM cells. This suggests that an acroterminal non-permissive or repellent signal may cause the final superficial divergence of the VPM.

A final issue is why part of the VPMms does not reach the target tuberal area and persists as an unique periventricular bridge that connects the VPM with the RM. The slowing of migration noted at E13.5 may relate to this phenomenon, unusual in the field of neuroembryology. Our data suggest so far that these incompletely migrated cells share molecular properties with the fully migrated ones, but analysis of adhesivity markers might discover some heterochronic differential aspects.

### Definition of RM and Diversity of Its Cell Populations

Assuming radial histogenesis as the fundamental pattern of hypothalamic nuclear development, Altman and Bayer (1986) postulated the neuroepithelium of the mamillary recess (sometimes identified in the literature as ‘tuberomamillary recess’) as the niche of VPM and DPM progenitors. Present data demonstrating their origin at the RM area accordingly falsate this conclusion. In recent years, various hypothalamic studies have highlighted a diversity of tangential migrations within, from or into the basal hypothalamus (Zhao et al., 2008; Morales-Delgado et al., 2011, 2014; Skidmore et al., 2008, 2012; Puelles et al., 2012; Díaz et al., 2015; Alvarez-Bolado, 2019; Murcia-Ramón et al., 2020), so that this possibility needs to be considered in neurogenetic studies.

We centered our attention on the RM area as a source of several tangential migrations (STh, PSTh, VPM, DPM), with emphasis on the VPM. The RM was originally defined in the rat as a ‘supramamillary area’ (Gurdjian, 1927). This term reflects usage of the classic columnar forebrain model (Herrick, 1910), in which the hypothalamus is the ventral/floor longitudinal zone or column of the diencephalon. M and RM figure in this model as ‘caudal hypothalamic’ regions (the postulated axis runs along the hypothalamus ending in the telencephalon; Figure 1B; Paxinos and Watson, 1998; Pan and McNaughton, 2004; Swanson, 2012). It is according to this classic forebrain model and axis that the VPM and DPM nuclei seem to be ‘premamillary’ (as well as ‘ventral’ and ‘dorsal,’ respectively), or the RM area appears to be ‘supramamillary.’ Within this alternative (and now obsolete) model, the VPM migration would proceed ventralwards, while the STh migration would advance rostralwards (compare Figures 1A,B). The present perimamillary/periretromamillary and ventral tuberal or retrotuberal bands were not identified in this classic schema, nor was it conceived originally that cells migrate tangentially ‘ventralwards’ from the ‘caudal’ hypothalamus region containing the mamillary and supramamillary areas into a theoretically *more rostral* tuberal hypothalamus region. To avoid confusion, we have retained the classic names of the relevant nuclei, excepting the RM concept corrected long-ago (Puelles et al., 1987); see additional comments about semantic problems within this brain territory in Puelles et al. (2012) and Puelles (2019).

In our present analysis we used instead as reference the modern updated prosomeric model of Puelles et al. (2012) and Puelles and Rubenstein (2015), whose length axis is orthogonal to the old columnar axis at hypothalamic levels (red line; Figures 1A,C). This conceptual revision stands primarily (1) on the analysis of longitudinal ventral midline gene markers which reflect early notochordal induction of the topologically longitudinal floor plate, strictly up to the mamillary floor (yellow; Figures 1A,C; Puelles et al., 2012, 2016; Puelles, 2013), and (2) on the fate-mapping demonstration of the rostral end of the fused neural tube longitudinal roof plate at the median crossing site of the anterior commissure (pink in Figure 1A; Puelles et al., 1987; Cobos et al., 2001). These two unique median neural longitudinal zones (floor and roof plates), already identifiable at neural plate stages, further represent the sources of ventralizing and dorsalizing forebrain diffusing signals (e.g., ventralizing SHH versus dorsalizing BMPs and WNTs). The antagonistic dorsoventral interaction of these patterning mechanisms secondarily establishes in the hypothalamus, as in the rest of the brain, the longitudinal alar and basal plates, and the related longitudinal alar-basal boundary (red line in Figure 1C). The latter is characterized modernly not by the variable sulcus limitans of His (due to tertiary protrusive morphogenesis of the basal plate rather than to patterning), but by a thin constant longitudinal band of expression of the markers *Nkx2.2*, *Nkx2.9*, *Ptc1*, and others, which emerges at the dorsoventral equilibrium site of dorsalizing versus ventralizing effects (Puelles et al., 2012; Puelles and Rubenstein, 2015). A number of other boundaries are interpreted as transversal (segmental or neuromeric) in this model when they are orthogonal to all three floor, alar-basal boundary and roof domains of the brain (Puelles and Rubenstein, 2003).

Our Figure 1C and its legend illustrate how the prosomeric RM is defined relative to surrounding longitudinal or transverse (neuromeric) domains of the hypothalamus, in a high-magnification map of hypothalamic neuromeres hp1 and hp2, centered on their basal progenitor domains (Puelles et al., 2012; Puelles and Rubenstein, 2015). The prosomeric model of the hypothalamus includes a novel rostromedian dorsoventral domain called the acroterminal area (blue in Figures 1A,C), which displays at its basal levels the anterobasal area, the median eminence, the median infundibulum of the neurohypophysis, the tuberomamillary recess and the rostromedian parts of the perimamillary and mamillary areas. The prosomeric model incorporates immediate patterning assumptions for the basal hypothalamus, insofar as the floor plate defined at the RM and M areas is a source of SHH and maybe other ventralizing morphogens. Basal ventralization can explain the pattern of topologically parallel longitudinal domains (e.g., TuD, TuI, TuV, PM, M, M floor; Figures 1A,C). In its turn, the acroterminal area, which is an apparent median source of *rostralizing* morphogens of the FGF family (Ferran et al., 2015; Puelles, 2017; Diaz and Puelles, 2020), can explain the rostrocaudal differences found between Tu and RTu, PM and PRM, or M and RM.

The fact that there exist four migrated RM derivatives (STh, PSTh, VPM, DPM), plus medial and lateral non-migrated RM nuclei (RMM, RML; Figures 2I,J) already suggests that RM probably is heterogeneous in terms of progenitor cell populations. Recent transcriptomic analysis of the adult VPM and RM populations has disclosed that given subgroups of cells have differential patterns of gene expression postnatally (Mickelsen et al., 2020). Theoretically, one way this differentiative property might begin is at the progenitor level, either via patterned dorsoventral and/or rostrocaudal subdivision of the initial RM field (i.e., via possible patterning and regionalizing roles of the floor and acroterminal organizers just mentioned), or via cell-to-cell inhibitory interactions among RM neuroepithelial cells (such as are described in the neural retina), leading to a salt-and-pepper uniformly distributed pattern of multiple distinct progenitors. Alternatively, a limited number of immature neuronal types may be produced in subareas of the RM, which thereafter migrate diversely (i.e., STh versus VPM directions), and only establish at a later postmigratory date further typological differentiations on the basis of epigenetic influences (e.g., trophism, local signals, retrograde signals collected by the axons). Importantly, most of the differential VPM gene markers discovered by Mickelsen et al. (2020) are first expressed after the VPM migration is finished, suggesting that the third possibility given above probably applies. At the present state of our knowledge, we cannot ascertain precisely which of these multiple adult phenotypes originate from *Foxa1* versus *Nr4a2* backgrounds (or arise from additional primary cell types not studied here). However, postnatally activated genes associated to the VPM core domain clearly include *Tac1*, *Nos1*, *Calb2*, and *Foxp2*, and, in our opinion, possibly also *Nr4a2*, *Slc6a3*, and *Dcd*, whereas the remaining genes of the Mickelsen et al. (2020) list apparently involve preferentially the shell subpopulation.

We observed initial differences in the expression of the transcription factors *Foxa1* and *Nr4a2* in the RM area, the VPMms and the emergent VPM nucleus. *Foxa1* signal predominates in ventral parts of RM, while *Nr4a2* is prevalent at corresponding dorsal parts (Figures 2A–F,G). This might imply a patterned initial *dorsoventral* subdivision of RM. Moreover, the apparent origin within RM of the VPMms involves particularly a rostral part of the dorsal half of RM, whereas the STh migration seems to start at the caudal parts of the RM. This implies a patterned *rostrocaudal* subdivision of the whole RM or of its dorsal half. For all we know, the RM area may be divided into four subdomains along the dorsoventral and anteroposterior dimensions. Note that apart the tangentially migrated populations this area also produces local medial, lateral and superficial cell populations, possibly derived from the ventral half of RM (RMM, RML; Puelles et al., 2012).

Once the periventricular VPMms starts at E12.5, the initially separate ventral and dorsal future VPM cells express mainly the corresponding markers; subsequently they mix together within the VPMms and the VPM (Figures 2E,F,F’,F”,F”’,G). Our *in vitro* CMFDA labeling experiments doubly counterstained with Foxa1 and Nr4a2 immunoreaction showed diverse types of labeling types along the VPMms stream: CMFDA + Foxa1 + Nr4a2 cells, CMFDA + Foxa1 cells, CMFDA + Nr4a2 cells, and neurons only showing CMFDA label (Figure 11). From this pattern it may be deduced, firstly, that, as regards *Foxa1* and *Nr4a2*, there may be three dorsoventral areal subdivisions of RM, rather than two, where the intermediate one produces the doubly labeled CMFDA + Foxa1 + Nr4a2 elements. Such double-labeled cells are also present within the VPM, side-by-side with the single-labeled ones (e.g., yellow-fluorescent cells in Figure 2G). At E18.5, the VPM clearly shows core subpopulations of Nr4a2 + and Foxa1 + /Nr4a2 + neurons, surrounded by a selectively Foxa1 + shell population which reaches the superficial tuberal stratum (Figures 2I,I’,J,J’). Secondly, the migrating elements labeled only with CMFDA in our doubly counterstained experiments indicate the existence of at least a fourth separate RM source of migrating cells, whose molecular profile does not include either *Foxa1* or *Nr4a2*. This possibly corresponds to a so far unrecognized rostrocaudal subdivision of RM, unless it relates instead to a caudal mamillary area origin (note most VPMms labeled cells originate rostrally within RM, and our experiments sometimes broached upon the adjacent M area; Figure 7). More discriminative experiments are needed to resolve this point.

Overall, this analysis allows us to conclude that there are data supporting the separate origin within RM of at least four molecularly different cell types which participate in the VPMms phenomenon.

Within the prosomeric model, the DPM nucleus paradoxically lies (topologically) *ventral* to the VPM (Figures 1A,B). The DPM forms within the terminal perimamillary band (PM; Figures 1G,H; Puelles et al., 2012) and may contain both intrinsic and migrated cells. There are genes shared among VPM and DPM (e.g., *Nr4a2*, *Bcl11a*, *Foxp1*, *Enc1*, *Pknox2*, apart of others related to their common glutamatergic profile; Puelles et al., 2012), whereas other markers are only present either in VPM (Supplementary Table 2), or in DPM (e.g., *Sim1*, *Fezf2*, *Ebf3*; Puelles et al., 2012; Allen Developing Mouse Brain Atlas; *Dlk1*, *Synpr1*, *Stxbp6*; Mickelsen et al., 2020). Some of our descriptive data indicate a contribution of Nr4a2 positive (but Foxa1 negative) RM neurons to the DPM (Figures 3G,H,K,M,N, 5A,B).

The dorsalward migration of the STh/PSTh population which apparently begins in a caudal part of the RM area is well studied. Marchand (1987), using the columnar model, located the progenitor zone of these neurons in the ‘caudal’ hypothalamic neuroepithelium, ‘dorsal’ to the mamillary recess, which clearly refers to our prosomeric RM area. These neurons make a subpial dorsalward migration into the intermediate retrotuberal domain, in an area close to the hypothalamo-diencephalic boundary that is covered later by the descending peduncle (lateral forebrain bundle), just before it bends caudalwards into the diencephalic tegmentum (Puelles et al., 2012). Mice with deficiencies in *Pitx2* and *Foxa1* transcription factors were separately shown to fail in this migration (Martin et al., 2004; Gasser et al., 2016). Although both VPM and STh migrating populations express some common markers such as *Foxa1*, *Lmx1b* and others, *Pitx2* is selective of the STh/PSTh complex (Allen Data). Furthermore, the RM area autochthonously gives rise to the local non-migrating medial and lateral RM nuclei which have differential projections to the hippocampal region. The STh nucleus participates instead in the direct basal ganglia circuit for motor control. The VPM is involved in the regulation of metabolic, olfactory and reproductive cues and shows connectivity with the amygdala complex, the septum, the preoptic area and the paraventricular hypothalamic nucleus, among others (Canteras et al., 1992). The divergence in the adult anatomical positions, connectivity and functions of these nuclei bespeaks of *primary heterogeneity* in their areal sources within RM. The relative positions and timing of their neurogenesis might be key factors in the specification of differently fated neurons originating from the RM neuroepithelial region.

### VPM Migration in the Context of the Prosomeric Model

As previously mentioned, the prosomeric model establishes a division of the hypothalamic basal plate into five longitudinal domains, RM and M building jointly the most ventral subdivision.

Some studies attribute a “similar” origin to the M and RM nuclei (Fu et al., 2017; Kim et al., 2020) without referring to their differential neuromeric identity (Puelles et al., 2012; Puelles and Rubenstein, 2015). Other works (e.g., Marion et al., 2005; García-Calero et al., 2008; Heide et al., 2015) consider a selective differentiation of the mamillary bulges involving genes not expressed in the RM area. One of the prosomeric criteria that distinguish the RM and M areas is the differential molecular profile of the hypothalamic floor plate in these regions. The RM floor expresses *Lmxb1* and *Foxa1*, whereas the M floor is negative for these two markers and expresses selectively *Foxb1*.

Parallel to the RM origin of the *Foxa1*-positive STh/PSTh migration, there is a mamillary origin of *Foxb1*-positive neurons of the newly discovered Parvafox nucleus (Zhao et al., 2008; Bilella et al., 2014, 2016). These cells migrate subpially dorsalward from M into the alar paraventricular area, always within the terminal hypothalamus (Puelles et al., 2012). Both STh/PSTh and Parvafox migrations follow strictly a topological dorsalward path, advancing toward their destination within the prosomere in whose ventral basal plate they were born.

This neighboring parallelism contrasts with the VPM migration described here, since this crosses *en masse* the intrahypothalamic interneuromeric limit (hp1/hp2), forming an oblique periventricular corridor over the M area and across the perimamillary area, into its tuberal destination within the rostrally adjacent prosomere but remaining within the basal plate. Our control labeling experiments of the mamillary area did not elicit any migration approaching the VPM (or the DPM).

The oblique rostralward course of the VPM migration is therefore a result whose explanation within the dorsoventral and rostrocaudal dimensions of the prosomeric model possibly requires the conjunction of a feeble dorsalward vector and a stronger rostralward vector. Any of these vectors may be an attraction or a repulsion.

Aiming to explore some theoretic possibilities, we examined at E16.5 and E18.5 conditional KO embryos of Ephrin-B2 activated at E10.5, but did not find evidence of a phenotype in our area of interest. Similarly, we studied E16.5 embryos of either Netrin1^–/–^ or DCC^–/–^ mutants, which also lacked a phenotype affecting the VPM migration. We finally examined severe Fgf8^neo/null^ hypomorphs at E18.5, given that the corresponding gene is expressed at the basal acroterminal midline rostral to the mamillary and tuberomamillary regions (Ferran et al., 2015) and the RM area expresses the *Fgfr2* gene coding an FGF receptor (Allen Developing Mouse Brain Atlas); the FGF8 morphogen might diffuse into the RM source of the VPM migration. This material only showed some hypoplasic abnormalities in the area of interest, discussed in the next section.

### Fgf8 in VPM Development

In our phenotypic analysis of two available Fgf8^neo/null^ severe hypomorph specimens (with only 20% of the normal amount of Fgf8 protein) we found a general reduction in the cellularity of the RM, VPMms and VPM nucleus neurons, as well as of their STh/PSTh neighbors, mainly affecting the *Foxa1* population (less clear results were obtained for *Nr4a2* neurons). The respective migrations nevertheless occurred in the corresponding directions. This result suggest a possible trophic effect on *Foxa1* cell production and/or cell survival. The *Foxa1-*positive RM population is shaped in wildtype mice –in sagittal sections- as an “arc” which surrounds the mamillary body, as we showed at different mediolateral section levels. The Fgf8^neo/null^ animals have only a ventral remnant of RM *Foxa1* signal visible only in medial sagittal sections. Moreover, both VPMms and VPM are somewhat altered in their shapes, in addition to showing a reduced size (present at less section levels than in wildtype embryos). The *Foxa1* population is severely reduced in VPMms and VPM, so that only dispersed cells appear laterally at the place where the laterally divergent superficial part of VPM appears in wildtype mice.

Various studies have remarked on a role of FGF8 in the specification of neuroepithelial domains via regulation of transcription factors, for instance in the telencephalon (Garel et al., 2003; Storm et al., 2006; Sato et al., 2017), thalamus (Botella-López et al., 2019), and isthmus (Liu et al., 1999; Martinez et al., 1999). The action of FGF8 in the maintenance of proliferation and/or repression of apoptosis at certain brain areas is well established (Kawauchi et al., 2005; Storm et al., 2006; Chung et al., 2016; Botella-López et al., 2019). Also, Tsai et al. (2011) advanced comments about a possible role of FGF8 in neural migration but it is not clear whether this is a direct or indirect consequence of its positive action on cell proliferation and axon outgrowth.

Some previous studies showed alterations in the pituitary and neuropeptide producing areas in the basal tuberal hypothalamus of Fgf8 hypomorphs (Brooks et al., 2010; McCabe et al., 2011; Rodriguez et al., 2015). It is not surprising that FGF8 has a role in the developing hypothalamus due to its high level of acroterminal expression (jointly with other Fgf family members; see Ferran et al., 2015; Diaz and Puelles, 2020), matching with Fgf8 receptors, mainly Fgfr2, distributed throughout the hypothalamic basal plate. Even though reduced in its cellularity, a recognizable VPM nucleus appears in the hypoplasic Tu area at E18.5 (present results). Therefore, some tangential migration happened in Fgf8^neo/null^ mice having FGF8 levels as low as 20% of the levels found in wildtype mice. There is no ectopic cell accumulation that might indicate a direct disturbance or aberrant route of the VPM migration in the mutant. The strong size reduction of the *Foxa1*-positive RM zone may indicate diminished proliferation, or excessive apoptosis of immature RM cell populations, which could explain the distorted shape and volume of both the VPMms/VPM and the STh/PSTh complexes. These results obviously do not resolve the mystery of the mechanism guiding this migration.

## Conclusion

Our mouse data support the conclusion that the neuronal population of the VPM is molecularly heterogeneous, originates at E12.5 and E13.5 from the rostrodorsal part of the retromamillary hypothalamic area (ventrobasal peduncular hypothalamus), and migrates tangentially rostralwards (mainly during E12) via a *periventricular* oblique course that contours dorsally the mamillary nucleus, crosses the overlying Otp/Sim1-positive perimamillary band and the thin tuberomamillary area, and penetrates the intermediate/ventral tuberal area, stopping short of the basal acroterminal area after deviating radially toward the tuberal surface (basal terminal hypothalamus). A majority of these migrating cells express either *Foxa1* or *Nr4a2*, or co-express both markers, while *non-identified* markers (or marker) apparently would characterize an additional subset of jointly migrated cells (i.e., there are at least 4 primary groups of molecularly distinct neurons). Some data suggested a partial contribution of *Nr4a2*-expressing cells likewise to the dorsal premamillary nucleus, which forms *ventrally* to the VPM within the perimamillary band (note the classic DPM/VPM terms are *columnar* while the described topology is *prosomeric*, involving a different axis of reference, at a right angle; Figures 1A,B). The initial molecular heterogeneity of VPM precedes marked *postmigratory* differentiative diversification of functional markers recently described by single-cell transcriptomic analysis of this nucleus (Mickelsen et al., 2020). The migrating Foxa1 and Nr4a2 populations apparently arise from distinct RM subdivisions, the origin of Nr4a2 cells being relatively dorsal to that of Foxa1 cells (double-labeled cells in between). We also observed that low levels of local FGF8 signal do not impede the studied migration, but the cellularity of the whole basal hypothalamus is hypoplasic, including the RM and the subthalamic and ventral premamillary populations, bespeaking of a trophic effect of FGF8. Nevertheless, these numerically compromised nuclei still achieved a topographically well-oriented migration, suggesting that their guidance mechanism is still efficient at low levels of FGF8.

## Data Availability Statement

The original contributions presented in the study are included in the article, further inquiries can be directed to the corresponding author.

## Ethics Statement

The animal study was reviewed and approved by Directive 2010/63/EU, Royal Decree 1201/2005 and 53/2013 Law 32/107 University of Murcia Committee (No. A13170406).

## Author Contributions

LP conceived and designed the study. LL-G performed the experiments. AA provided some ISH images. LL-G, LP, and EG-C analyzed the data. LL-G and LP prepared the figures and the tables. EP provided the transgenic Fgf8 specimens. LL-G and LP wrote the manuscript. LP provided funding for this study. All the authors approved the final version submitted.

## Conflict of Interest

The authors declare that the research was conducted in the absence of any commercial or financial relationships that could be construed as a potential conflict of interest.

## Footnotes

1 https://developingmouse.brain-map.org/

## Abbreviations

A/B: alar basal boundary
Arc: arcuate nucleus
Cb: cerebellum
DM-P: peduncular part of the dorsomedial nucleus
DM-T: terminal part of the dorsomedial nucleus
DPM: dorsal premamillary nucleus
hp1: hypothalamic prosomere 1
hp2: hypothalamic prosomere 2
is: isthmus
ISH: *in situ* hybridization
M: mamillary area
M: mesencephalon
ME: medial eminence
Mtg: mamillotegmental tract
NHy: neurohypophysis
OCh: optic chiasma
Ot: optic tract
p1: prosomere 1
p2: prosomere 2
p3: prosomere 3
ped: peduncular tract
PHy: peduncular hypothalamus
PM: perimamillary area
PRM: periretromamillary area
PSTh: parasubthalamic nucleus
Pt: pretectum
PTh: prethalamus
r1: rhombomere 1
r2: rhombomere 2
RM: retromamillary area
RTuD: retrotuberal dorsal area
RTuI: retrotuberal intermediate area
RTuV: retrotuberal ventral area
SNc: substantia nigra pars compacta
STh: subthalamic nucleus
Th: thalamus
THy: terminal hypothalamus
TuD: tuberal dorsal area
TuI: tuberal intermediate area
TuV: tuberal ventral area
VM: ventromedial nucleus
VPM: ventral premamillary nucleus
VPMc: core or central part of ventral premamillary nucleus
VPMlat: shell or lateral part of ventral premamillary nucleus
VPMms: ventral premamillary migratory stream
ZLi: *zona limitans.*
