# Supplementary material for: Tangential Intrahypothalamic Migration of the Mouse Ventral Premamillary Nucleus and Fgf8 Signaling
Source: Front Cell Dev Biol. 2021 May 19;9:676121. doi: 10.3389/fcell.2021.676121 (PMC8170039; doi:10.3389/fcell.2021.676121)
Supplement: Supplementary file 1 [file Table_1.pdf]

Suppl. Table 1

ISH probes information

**Gene**	**NCBI accession number**
Ebf3	NM_001113414.1
Fezf2	NM_080433.3
Foxa1	NM_008259.4
Foxb1	NM_022378.3
Nr4a2	NM_013613.2
Sim1	NM_011376.3
