# Supplementary material for: Tangential Intrahypothalamic Migration of the Mouse Ventral Premamillary Nucleus and Fgf8 Signaling
Source: Front Cell Dev Biol. 2021 May 19;9:676121. doi: 10.3389/fcell.2021.676121 (PMC8170039; doi:10.3389/fcell.2021.676121)
Supplement: Supplementary file 2 [file Table_2.pdf]

Suppl. Table 2

VPM gene markers and their expression in retromamillary (RM), subthalamic nucleus (STh) and/or dorsal premamillary nucleus (DPM) at E13.5, E15.5, 18.5 and P14, according to Allen Developing Mouse Brain Atlas hybridization *in situ* data.

Gene	RM				VPM			STh complex			DPM		
	E13.5	E15.5	E18.5	P14	E15.5	E18.5	P14	E15.5	E18.5	P14	E15.5	E18.5	P14
Alcam	X	X	X	X	X	X	X						
Ar							X						X
Bcl11a	X	X	X		X	X		X	X		X	X	
Calb2	X	X	X	X		X	X		X	X		X	X
Foxa1	X	X	X	X	X	X	X	X	X	X			
Foxp1						X			X			X	
Foxp2	X	X	X	X	X	X	X	X	X	X			
Grik2	X	X	X		X	X			X		X	X	
Irs4					X	X	X						
Irx1	X	X			X	X	X						
Irx3	X	X			X	X	X						
Irx5	X	X			X	X	X						
LepR							X						
Lmx1a	X	X	X	X	X	X	X	X	X	X			
Lmx1b	X	X	X	X	X	X	X	X	X	X			
Nos1		X	X	X	X	X	X			X			X
Nr4a2	X	X	X	X	X	X	X				X	X	X
Pbx3	X	X	X		X	X	X		X	X			
Pknox2	X	X	X			X						X	
Satb1		X	X	X	X	X	X						
Slc6a3							X						
Tac1						X	X		X	X			X
