# Supplementary material for: Tangential Intrahypothalamic Migration of the Mouse Ventral Premamillary Nucleus and Fgf8 Signaling
Source: Front Cell Dev Biol. 2021 May 19;9:676121. doi: 10.3389/fcell.2021.676121 (PMC8170039; doi:10.3389/fcell.2021.676121)
Supplement: Supplementary file 3 [file Table_3.pdf]

Suppl. Table 3

Sorting of 79 organotypic cultures (E12.5-E14.5 mice brains) of CMFDA labelling .We indicate for each case: the serial number, the antibody/antibodies used (Nr4a2, Nr4a2/Otp or Nr4a2/Foxa1), the position of the CMFDA-tungsten particle out of retromamillary area (RM) at premamillary (PRM), mamillary (M), mamillary/retromamillary (M/RM) regions ; or within RM, at 4, 3, 2, 1 dorsoventral tiers, and caudal (C), middle (M), rostral (R), or VPMms rostrocaudal positions. Positive cases for VPMms -CMFDA labelling are indicated as “Pos”; negative cases for VPMms-CMFDA labelling are indicated as “Neg”. Number of cases marked outside RM: PRM (n=5), M (n=5), M/RM (n=10). Number of cases marked within RM: Position 4 (n=3), Position 3C (n= 2), Position 3M (n=3), Position 3R (n=12), Position 3VPMms (n=12), Position 2C (n=1), Position 2M (n=3), Position 2R (n=6), Position 2VPMms (n=8), Position 1C (n=1), Position 1M (n=3), Position 1R (n=2), Position 1VPMms (n=3).

Case number (E12.5-E14.5)	Immunofluorescence			PRM	M	M/RM	Pos. 4	Position 3				Position 2				Position 1				Pos	Neg
	Nr4a2	Nr4a2/Otp	Nr4a2/Foxa1				(RM)	C(RM)	M(RM)	R(RM)	VPMms	C(RM)	M(RM)	R(RM)	VPMms	C(RM)	M(RM)	R(RM)	VPMms		
C001	X								X												X
C002	X														X					X	
C006	X					X															X
C007	X				X																X
C008	X					X															X
C009	X					X															X
C011	X					X															X
C012	X						X														X
C013	X					X															X
C014	X									X										X	
C015	X					X															X
C017	X													X						X	
C018	X									X										X	
C019	X													X						X	
C020	X									X										X	
C021	X					X															X
C022	X				X																X
C023	X									X										X	
C024	X													X						X	
C025	X									X										X	
C026	X							X													X
C027	X									X										X	
C028															X					X	
C029	X										X									X	

Case number (E12.5-E14.5)	Immunofluorescence			PRM	M	M/RM	Pos4	Position 3				Position 2				Position 1				Pos	Neg
	Nr4a2	Nr4a2/Otp	Nr4a2/Foxa1				(RM)	C(RM)	M(RM)	R(RM)	VPMms	C(RM)	M(RM)	R(RM)	VPMms	C(RM)	M(RM)	R(RM)	VPMms		
C030	X												X							X	
C031	X					X															X
C033	X													X						X	
C034	X										X									X	
C035	X				X																X
C036	X										X									X	
C037	X										X									X	
C038	X				X																X
C039	X												X							X	
C040	X									X										X	
C041	X																	X		X	
C042	X										X									X	
C043	X										X									X	
C044	X										X									X	
C046	X									X										X	
C047	X										X									X	
C048	X														X					X	
C049	X									X										X	
C050		X									X									X	
C051		X					X														X
C052		X						X													X
C053		X												X						X	
C054		X							X												X
C055		X																	X	X	
C057		X									X									X	

Case number (E12.5-E14.5)	Immunofluorescence			PRM	M	M/RM	Pos4	Position 3				Position 2				Position 1				Pos	Neg
	Nr4a2	Nr4a2/Otp	Nr4a2/Foxa1				(RM)	C(RM)	M(RM)	R(RM)	VPMms	C(RM)	M(RM)	R(RM)	VPMms	C(RM)	M(RM)	R(RM)	VPMms		
C058		X							X												X
C059		X			X																X
C060		X									X									X	
C061		X					X														X
C062		X									X									X	
C063		X											X							X	
C064		X				X															X
C065		X								X										X	
C066		X								X										X	
C067		X				X															X
C068		X													X					X	
C069		X										X									X
C070	X																X			X	
C071	X			X																	X
C072	X															X					X
C073	X									X										X	
C074	X													X						X	
C075	X			X																	X
C076	X																		X	X	
C077	X																		X	X	
C078	X																	X		X	
C079	X			X																	X
C080	X			X																	X
C082	X																X			X	
C083	X																X			X	

Case number (E12.5-E14.5)	Immunofluorescence			PRM	M	M/RM	Pos4	Position 3				Position 2				Position 1				Pos	Neg
	Nr4a2	Nr4a2/Otp	Nr4a2/Foxa1				(RM)	C(RM)	M(RM)	R(RM)	VPMms	C(RM)	M(RM)	R(RM)	VPMms	C(RM)	M(RM)	R(RM)	VPMms		
C084	X			X																	X
C085			X												X					X	
C086			X												X					X	
C087			X												X					X	
