# Supplementary material for: Tangential Intrahypothalamic Migration of the Mouse Ventral Premamillary Nucleus and Fgf8 Signaling
Source: Front Cell Dev Biol. 2021 May 19;9:676121. doi: 10.3389/fcell.2021.676121 (PMC8170039; doi:10.3389/fcell.2021.676121)
Supplement: Supplementary file 4 [file Table_4.pdf]

Suppl. Table 4

Sorting of 17 organotypic cultures (E13.5-E15.5 mice brains) of CMFDA labelling. We indicate for each case: the serial number, the antibodies used (Nr4a2/Otp), the position of the CMFDA-tungsten particle within RM at 4, 3, 2, 1 dorsoventral tiers, and caudal (C), middle (M), rostral (R), or VPMms rostrocaudal positions. Positive cases for VPMms -CMFDA labelling are indicated as “Pos”; negative cases for VPMms-CMFDA labelling are indicated as “Neg”. Asterisks indicate reduced CMFDA cell amount along VPMs without PM crossing. Number of cases: Position 3M (n=1), Position 3R (n=3), Position 3VPMms (n=4), Position 2M (n=2), Position 2R (n=2), Position 2VPMms (n=1), Position 1M (n=2), Position 1R (n=1), Position 1VPMms (n=1).

Case number (E13.5-E15.5)	Immunofluorescence	Position 3				Position 2				Position 1				Pos	Neg
	Nr4a2/Otp	C(RM)	M(RM)	R(RM)	VPMms	C(RM)	M(RM)	R(RM)	VPMms	C(RM)	M(RM)	R(RM)	VPMms		
C089	X						X							X*	
C090	X											X			X
C091	X								X					X	
C092	X							X						X*	
C093	X										X			X*	
C094	X				X									X	
C095	X						X							X	
C096	X		X												X
C097	X			X										X	
C098	X			X										X*	
C099	X				X										X
C100	X										X			X	
C101	X				X									X*	
C102	X				X									X	
C103	X							X						X	
C104	X												X	X	
C105	X			X										X	
